# High-quality *Momordica balsamina* genome elucidates its potential use in improving stress resilience and therapeutic properties of bitter gourd

**DOI:** 10.3389/fpls.2023.1258042

**Published:** 2024-01-24

**Authors:** N. D. Vinay, Kalpana Singh, Ranjith Kumar Ellur, Viswanathan Chinnusamy, Sarika Jaiswal, Mir Asif Iquebal, Anilabha Das Munshi, Hideo Matsumura, G. Boopalakrishnan, Gograj Singh Jat, Chittaranjan Kole, Ambika Baladev Gaikwad, Dinesh Kumar, Shyam Sundar Dey, Tusar Kanti Behera

**Affiliations:** ^1^Division of Vegetable Science, Indian Council of Agricultural Research (ICAR)-Indian Agricultural Research Institute, New Delhi, India; ^2^Division of Agricultural Bioinformatics, Indian Council of Agricultural Research (ICAR)-Indian Agricultural Statistics Research Institute, New Delhi, India; ^3^Division of Genetics, Indian Council of Agricultural Research (ICAR)-Indian Agricultural Research Institute, New Delhi, India; ^4^Division of Plant Physiology, Indian Council of Agricultural Research (ICAR)-Indian Agricultural Research Institute, New Delhi, India; ^5^Gene Research Centre, Shinshu University, Ueda, Japan; ^6^Foundation for Science and Society, Kolkata, India; ^7^Division of Genomic Resources, Indian Council of Agricultural Research (ICAR)-National Bureau of Plant Genetic Resources, New Delhi, India; ^8^Indian Council of Agricultural Research (ICAR)-Indian Institute of Vegetable Research, Varanasi, Uttar Pradesh, India

**Keywords:** cucurbits, genome assembly, genomic resources, medicinal value, *Momordica balsamina*, stress tolerance

## Abstract

**Introduction:**

*Momordica balsamina* is the closest wild species that can be crossed with an important fruit vegetable crop, *Momordica charantia*, has immense medicinal value, and placed under II subclass of primary gene pool of bitter gourd. *M. balsamina* is tolerant to major biotic and abiotic stresses. Genome characterization of *Momordica balsamina* as a wild relative of bitter gourd will contribute to the knowledge of the gene pool available for improvement in bitter gourd. There is potential to transfer gene/s related to biotic resistance and medicinal importance from *M. balsamina* to *M. charantia* to produce high-quality, better yielding and stress tolerant bitter gourd genotypes.

**Methods:**

The present study provides the first and high-quality chromosome-level genome assembly of *M. balsamina* with size 384.90 Mb and N50 30.96 Mb using sequence data from 10x Genomics, Nanopore, and Hi-C platforms.

**Results:**

A total of 6,32,098 transposons elements; 2,15,379 simple sequence repeats; 5,67,483 transcription factor binding sites; 3,376 noncoding RNA genes; and 41,652 protein-coding genes were identified, and 4,347 disease resistance, 67 heat stress–related, 05 carotenoid-related, 15 salt stress–related, 229 cucurbitacin-related, 19 terpenes-related, 37 antioxidant activity, and 06 sex determination–related genes were characterized.

**Conclusion:**

Genome sequencing of *M. balsamina* will facilitate interspecific introgression of desirable traits. This information is cataloged in the form of webgenomic resource available at http://webtom.cabgrid.res.in/mbger/. Our finding of comparative genome analysis will be useful to get insights into the patterns and processes associated with genome evolution and to uncover functional regions of cucurbit genomes.

## 1 Introduction

*Momordica balsamina* (2n = 2x = 22), commonly referred to as Balsam apple, Southern Balsam pear, or African pumpkin, is a wild plant belonging to the *Momordica* genus within the *Cucurbitaceae* family (Bharathi and John, 2013). It is native to tropical regions of Africa, Asia, and Australia (Jeffrey, 1967; Mishra et al., 1986). *M. balsamina* has an annual to perennial life cycle and grows as a trailing herb (John, 2005; Behera et al., 2010). It grows better in hot, humid climates and prefers acidic soil (pH 5.0–6.5) (Mishra et al., 1986). Ellipsoid-shaped immature fruits of *M. balsamina* are rich in essential vitamins (A and C) and vital minerals (iron and calcium) (Wehner et al., 2020). Additionally, its leaves are abundant in carotenoids (Mashiane et al., 2022). These nutritionally and medicinally enriched fruits and leaves are consumed in rural areas of Africa and Asia (Flyman and Afolayan, 2007; Bharathi and John, 2013). It is one of the four *Momordica* species cultivated in India, primarily in the dry regions of the Northwest plains, Eastern Ghats, and Western Ghats (Peter and Abraham, 2007).

Balsam apple has the reputation of a “gifted plant” due to its richness in bioactive compounds, which offer diverse therapeutic benefits. These compounds exhibit wide spectrum of medicinal values, including anti-septic, anti-microbial, anti-bacterial, anti-viral (including anti-HIV), anti-inflammatory, anti-plasmodial, antioxidant, and analgesic properties (Hassan and Umar, 2006; Thakur et al., 2009). The extensive range of medicinal properties exhibited by *M. balsamina* can be attributed to its diverse array of terpenoid compounds, such as balsaminol, balsaminoside, balsaminagenins, karavilagenin, cucurbalsaminol, and balsaminapentaol (Ramalhete et al., 2009; Ramalhete et al., 2010; Ramalhete et al., 2011a; Ramalhete et al., 2011b). Numerous researches have been conducted on these compounds in order to highlight their potential medical uses. Additionally, “cucurbitacins” derived from *M. balsamina* were found to have selective antiproliferative activity against multidrug resistant cancer cells (Ramalhete et al., 2022). Furthermore, Balsam apple contains ribosomal-inactivating proteins (RIPs) such as Momordin II and Balsamin, which possess remarkable antiviral, anticancer, and antibacterial properties. These RIPs have found practical applications in the development of commercial drug preparations (Khare, 2007; Kaur et al., 2012; Ajji, 2016; Ajji et al., 2017). The findings from these aforementioned studies justify the immense potential of *M. balsamina* within the pharmaceutical industry, thus making it a subject of intense scientific research in the field of cucurbitaceous vegetable crops.

*Momordica charantia*, commonly known as Bitter gourd, is the most widely cultivated vegetable within the *Momordica* genus, renowned for its distinctive bitter taste, attributed by cucurbitane-type tri-terpenoids (Chen et al., 2005). The fruits of Bitter gourd are abundant in vitamin C and iron and exhibit high antioxidant activity (Behera et al., 2010). Beyond its culinary use, it finds extensive application in traditional medicine, alleviating stomach pain, anemia, malaria, coughs, and fever, and it is a renowned source of anti-diabetic drug in pharmaceutical industry (Tan et al., 2008; Krawinkel et al., 2018). Despite its biological and economic significance, the crop improvement and varietal development program in Bitter gourd have been hindered by the limited genetic diversity found in natural populations (Dhillon et al., 2016). Furthermore, bitter gourd, being a crop of tropics and subtropics, is affected by various biotic and abiotic stresses. To overcome these obstacles, there is a critical need for diverse and valuable genetic resources to facilitate the development of elite high-yielding and resilient bitter gourd varieties (Cui et al., 2020).

Among the seven *Momordica* species found in India, *M. charantia* and *M. balsamina* are the only two species with monoecious sex expression. These two species share same basic chromosome number of *x* = 11 and exhibit similar frequencies and ranges of bivalent and chiasmata formation. This high karyo-morphological similarity indicates a close ancestral relationship between these two species (Trivedi and Roy, 1972; Singh, 1990; Bharathi et al., 2011). *M. balsamina*, in particular, is considered the closest wild relative that can be crossed with Bitter gourd, falling under the II subclass of the primary gene pool of Bitter gourd (Bharathi et al., 2012). *M. balsamina* also possesses a high level of tolerance to like pests such as ladybird beetle (*Epilacna septima*), pumpkin caterpillar (*Margaronia indica*), red pumpkin beetle (*Aulocophora fevicoli*), gall fly (*Lasioptera falcata*), root-knot nematode (*Meladogyne incognita*), and diseases such as yellow mosaic and little leaf disease, making it an invaluable genetic resource for the improvement of *M. charantia* (Rathod et al., 2021). Hence, in addition to medicinal attributes, *M. balsamina* can serve as a potent genetic source of biotic stress resistance.

Interspecific hybridization has proven to be a successful method for harnessing natural genetic variation and transferring desirable genes from wild relatives to cultivated crops (Bowley and Taylor, 1987; Dempewolf et al., 2017). In the Cucurbitaceae family, successful inter-specific hybrids have been developed within and between wild and cultivated taxa (Weeden and Robinson, 1986; Singh, 1991; Robinson and Decker-Walters, 1997). Likewise, there is great potential for the transfer of beneficial genes from *M. balsamina* to *M. charantia* for the genetic improvement of Bitter gourd. Previous studies have reported partial cross-compatibility between *M. charantia* and *M. balsamina*, resulting in progenies exhibiting normal meiosis (Singh, 1990; Bharathi et al., 2012). Recently, a detailed study on crossability involving 116 diverse Bitter gourd genotypes demonstrated success in six cross-combinations (Rathod et al., 2021). The study also confirmed the partial introgression of chromosome segments from *M. balsamina* into the Bitter gourd genome through morpho-cytological and molecular analysis of interspecific hybrids between *M. charantia* cv. Pusa Aushadhi × *M. balsamina* and their advanced generations (*F*_2_ and backcross generations). These findings suggest the possibility of transferring genes or traits related to biotic resistance and medicinal properties from *M. balsamina* to *M. charantia*, producing high-quality and resistant Bitter gourd varieties.

The era of genomics-assisted vegetable breeding commenced with the completion of the cucumber whole genome assembly in 2009 (Huang et al., 2009). In 2016, the first draft genome of Bitter gourd was published (Urasaki et al., 2017), followed by subsequent high-quality, chromosome-level assemblies (Cui et al., 2020; Matsumura and Urasaki, 2020). With advancements in sequencing technologies and bioinformatics tools, genomic data for flowering plants has been expanding rapidly (Chen et al., 2018), and genome assemblies for most cultivated cucurbits are now available in the public domain. Presently, there is a focus on genome characterization of closely related cross-compatible crop wild relatives (CWRs).

CWRs serve as a dynamic gene pool to access vital genetic diversity needed for crop improvement. Earlier, molecular techniques were used to characterize CWR (Dillon et al., 2007a; Sotowa et al., 2013). Now, advanced next-generation sequencing (NGS) platforms can be utilized for genome characterization of CWR to study phylogeny and discover useful genes in order to support agriculture and food security (Brozynska et al., 2016). Several wild relatives of tomato (Sato et al., 2012), brinjal (Gramazio et al., 2019), potato (Aversano et al., 2015), and sweet potato (Wu et al., 2018) have already been sequenced. In the current study, we present first high-quality genome assembly of *M. balsamina a*, close relative of bitter gourd that can be a vital genetic resource to improve medicinal value and stress resistance in bitter gourd.

## 2 Material and method

### 2.1 Sample collection and DNA extraction

Young leaf samples of *M. balsamina* (IC-467683) weighing around 10 g were collected for DNA isolation from 30-day-old seedlings at the active vegetative stage during the early morning hours. The collected leaf samples were packed immediately in aluminium foil, frozen into liquid nitrogen and stored at −80°C. Total DNA was isolated using the modified cetyl trimethyl ammonium bromide (CTAB) method (Saghai-Maroof et al., 1984). The genomic DNA samples were adjusted to 50 ng DNA/µL and stored at 4°C until used for sequencing. The quality and quantity of the extracted DNA were estimated with an Eppendorf Biospectrometer confirmed by running on 0.8% w/v agarose gel.

### 2.2 10x genomics sequencing and library preparation

High-molecular weight DNA (1.25 ng) was loaded onto a Chromium Controller chip, along with 10x Chromium reagents and gel beads following manufacturers recommended protocols. Initial library construction occurred within droplets containing Gel Beads-in-Emulsion (GEMs) beads with unique barcodes. The library construction incorporated a unique barcode adjacent to read one. All molecules within a GEM got tagged with the same barcode. However, because of the limiting dilution of the genome (roughly 300 haploid genome equivalents), the probability that two molecules from the same region of the genome were partitioned in the same GEM was minimal. Thus, the barcodes were used to associate short reads with their source long molecule statistically. The resulting library was sequenced on Illumina HiSeq X Ten sequencer (San Diego, CA, USA) as per the manufacturer’s protocol to produce 2 × 150 paired-end sequences. The entire process was performed on four replicates; thus, four pair-end libraries were prepared.

### 2.3 NanoPore sequencing and library preparation

First, 05-µg genomic DNA was sheared to approximately 15,000 bp by centrifugation at 5,200 rpm in a gTUBE. DNA was repaired with damage repair reagent and end-repaired using end-repair mix before ligation to nanopore blunt end adapter. Unligated material was digested with Exo III and Exo VII. Then, 12–25 Kb library fragments were purified via two consecutive Ampure cleanups, and size selection was done on Blue Pippin (SageScience, Beverley, MA, USA) with a 0.75% agarose cassette. An aliquot of 20 picomol of the final library was loaded onto the flow cell and sequenced on machine MinION (Oxford Nanopore Technologies, Oxford Science Park, United Kingdom) using Oxford Nanopore sequencing kit 2.0 and improved instrument workflow (Instrument Control Software 4.0).

### 2.4 Hi-C sequencing and library preparation

Fresh and young leaf samples were collected and cross-linked for 10 min with a 1% final concentration of fresh formaldehyde and quenched with a 0.2 M final concentration of glycine for 5 min. The cross-linked cells were subsequently lysed in lysis buffer. The extracted nuclei were re-suspended with a 150-µL 0.1% Sodium dodecyl sulfate (SDS) and incubated at 65°C for 10 min. Furthermore, they were quenched by adding 120 µL of water and 30 µL of 10% Triton X-100 and incubated at 37°C for 15 min. The DNA in the nuclei was digested by adding 30 µL of 10x NEB buffer 2.1 and 150 U of Mbol and incubated at 37°C for 12h. This was followed by inactivation of Mbol enzyme at 65°C for 20 min and filling of cohesive ends by adding 1 µL of each 10 mM deoxythymidine triphosphate (dTTP), deoxyadenosine triphosphate (dATP), and deoxyguanosine triphosphate (dGTP), 2 µL of 5 mM biotin-14-deoxycytidine triphosphate (dCTP), and 4 µL (40 U) Klenow and after that incubated at 37°C for 2h. To start proximity ligation, 120 pL 10x blunt-end ligation buffer, 100 pL 10% Triton X-100, and 20U T4 DNA ligase were added and held at 16°C for 4h. This was followed by reversing of the cross-linking with 200 ug/mL proteinase K (Thermo Fisher Scientific) at 65°C for 12h. Furthermore, chromatin DNA manipulations were performed using a method described by Belaghzal et al. (2017), followed by DNA purification using QIAamp DNA Mini Kits (Qiagen) and shearing of purified DNA in length of 400 bp. Dynabeads MyOne Streptavidin C1 (Thermo Fisher Scientific) was used to pull down point ligation junctions. NEB Next Ultra II DNA library Prep Kit for Illumina (NEB) was used to prepare Hi-C library for Illumina sequencing. The final library was sequenced on the Illumina HiSeq X Ten platform (San Diego, CA, USA) as per the manufacturer’s protocol with 2 × 150 paired-end mode.

### 2.5 Data pre-processing and genome assembly

**Figure 1:**
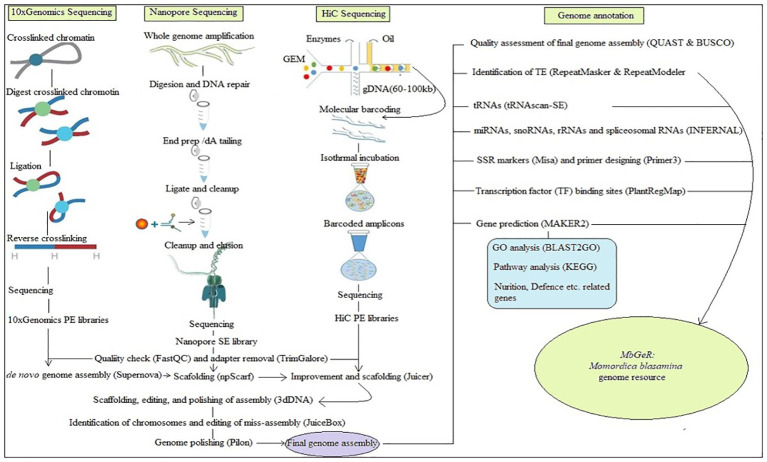
Over-view of pipeline adopted in the study.

All the raw reads of 10x Genomics, Nanopore and HiC libraries used in the present study have been submitted in National Center for Biotechnology Information (NCBI) with SRA IDs SRR21495983, SRR21495982, and SRR21495981, respectively. **Figure 1** shows the outline followed during the present study. Prior to assembly, reads of these libraries were cleaned using FastQC (http://www.bioinformatics.babraham.ac.uk/projects/fastqc: Andrews, 2010) by removing low quality reads at < 20 phred score, followed by adapter cleaning using TrimGalore (https://www.bioinformatics.babraham.ac.uk/projects/trim_galore/). *De-novo* genome assembly was performed using all the 10x Genomics libraries of four replicates using Supernova v2.1.1 (Weisenfeld et al., 2017). After this, Nanopore libraries were mapped on *de-novo* genome assembly for further scaffolding using npScarf (Cao et al., 2017). Finally, HiC libraries were mapped on improved genome assembly using Juicerv1.5 (Durand et al., 2016) to obtain the de-duplicated alignment file. Furthermore, scaffolding, editing, and polishing of assembly was performed using 3dDNA v180419 (Dudchenko et al., 2017). Finally, identification of chromosomes and editing of miss-assembly was performed using JuiceBox v1.11.08 (Robinson et al., 2018) to construct contact maps for chromosomes. Genome polishing was performed on final assembly using Pilon (Walker et al., 2014).

### 2.6 Validation of chromosome level assembly

To assess the quality of the assembled genome, assembly statistics were calculated using QUAST (Gurevich et al., 2013). Furthermore, validation of assembly was performed using BUSCO (Simao et al., 2015) to find the completeness and contamination within genome assembly. A comparative study of *M. balsamina* genome assembly with other related species, such as *Momordica charantia*, *Citrullus lanatus*, *Cucumis sativus*, and *Cucumis melo* was also performed.

### 2.7 Genome annotation

For genome annotation, a series of bioinformatics tools were employed. First, repeat regions of the assembled genome were masked using RepeatMasker v4.1.0 (http://www.repeatmasker.org/RMDownload.html). This was followed by the identification of transposable elements (TEs) using RepeatModeler (http://www.repeatmasker.org/RepeatModeler/) to find LINE, SINE, Simple Repeats, LTR elements, DNA elements, and so forth. The ncRNA-encoding genes were also identified from the assembled genome. Furthermore, tRNAs were identified using tRNA scan-SE v.1.3.1 (Chan and Lowe, 2019) with < 1 false positive per 15 gigabases. Other ncRNAs, such as microRNAs, snRNAs, rRNAs, and spliceosomal RNAs, were also identified using INFERNAL v1.1.4 (Nawrocki and Eddy, 2013) at default parameters., Protein-encoding genes were predicted using SEQing v0.1.45 (Lewinski et al., 2020), which is an automated pipeline of self-trained hidden Markov models (HMM) models and transcriptomic data for gene prediction by Glimmer HMM, SNAP, and AUGUSTUS and combining their results by MAKER2 in association with transcriptomic evidence of *Momordica charantia*. Finally, the predicted genes passed through Cluster Database at High Identity with Tolerance (CD-HIT) (Limin et al., 2012), clustering at 90% sequence similarity to extract non-redundant genes. Extraction of Single Sequence Repeat (SSR) markers was performed using MIcroSAtellites (MISA) (Beier et al., 2017), considering mononucleotide repeats motif with at least 10 repeats, dinucleotide with six, tri-, tetra-, penta-, and hexa-nucleotide with five repeats (Thiel et al., 2003). Compound microsatellites were defined as those with the interval between two repeats motifs ≤100 nucleotides in the previous reports (Zhao et al., 2017). Furthermore, primers were also designed for each of the SSR makers using Primer3 (Untergasser et al., 2012) with parameters 18–27 bp primer length, 57°C–63°C melting temperature, 30%–70% GC content, and 100–300 bp product size. Transcription factor (TF) binding sites were extracted using PlantRegMap (Jin et al., 2017).

### 2.8 Functional annotation of protein-coding genes

The predicted protein-coding genes were mapped against the NR database (updated May 2020) and the plant TF database (version 5.0) using NCBI blast (version 2.2.29+) (Lipman and Pearson, 1985) for functional annotation. Furthermore, gene ontology (GO) analysis was performed on predicted genes using Blast2GO (Conesa et al., 2005). Pathway analysis was performed using Kyoto Encyclopedia of Genes and Genomes (KEGG) pathways (Erxleben and Grüning, 2020).

### 2.9 Disease resistant, defence, stress, and sex expression–related genes

Disease resistant genes were identified by mapping proteins against the PRGDB database v.4.0 (García et al., 2021) with e-value cutoff of 1e-10 using BLAST (NCBI 2.2.29+) (Lipman and Pearson, 1985). Along with resistance genes, genes related to salt stress, heat stress and sex expression were also extracted.

### 2.10 Orthologous genes, phylogenetic, and synteny analysis

*M. balsamina* genes, orthologous with *M. charantia*, *Citrullus lanatus*, *Cucumis sativus*, and *Cucumis melo*, were predicted using OrthoMCL (Chen et al., 2006) based on a Markov Cluster algorithm to group (putative) orthologs utilizing all-against-all BLAST (Lipman and Pearson,1985) comparisons among protein sequences of considered species. For the detection of synteny between *M. balsamina* genome assembly and four other genome assemblies of abovementioned species was performed by SyMAP v4.2 (Soderlund et al., 2011). Synteny blocks shown as colored ribbons between the chromosomes arranged in circle were visualized in the circular plots using Circos (Krzywinski et al., 2009). Furthermore, micro-synteny, a synteny in small regions, identified between two or more genomic regions was performed between of *M. balsamina* and *M. charantia* genomes using McScan python version (Tang and Krishnakuar, 2015). Furthermore, a phylogenetic study was also performed among genome assemblies of *M. balsamina*, *M. charantia*, *Cucumis melo*, *Citrullus lanatus*, and *Cucumis sativus*. First, a multi-sequence alignment (MSA) was performed among genome assemblies using Multiple Alignment using Fast Fourier Transform (MAFFT) (Katoh et al., 2002). Later, a distance matrix was calculated among assemblies using MSA with Poisson correction method, >70% site coverage, and <30% alignment gaps, missing data, and ambiguous bases by ClustalW2 (Thompson et al., 1994). Finally, a phylogenetic tree was constructed using Neighbor-Joining method by ClustalW2.

### 2.11 Development of *M. balsamina* genomic resource

A web-genomic resource for *M. balsamina*, named MbGeR, was developed using all the results obtained from the genomic data analyses performed in the present study. MbGeR catalogs the information related to molecular markers such as SSRs, transposons elements (TEs), TF sites, ncRNAs and genes. It is based on a three-tier architecture, namely, client tier, middle tier, and database tier, developed using PHP, MySQL, HTML, and Apache. Web pages are developed using PHP and HTML in order to browse MbGeR and put up queries by users in client tier. All the information regarding transcripts, Differentially Expressed Genes (DEGs), markers, and so forth. are placed in different tables in MySQL database in the database tier. The scripting of client query page was done in PHP and HTML for execution and fetching in the middle tier. The web hosting was performed using Apache server. The bitter melon web resources are available at http://webtom.cabgrid.res.in/mbger/.

## 3 Result

### 3.1 Data pre-processing, genome assembly, and comparative analysis

In the present study, the whole genome of *M. balsamina* was assembled using reads obtained from three different platforms: Oxford Nanopore, 10 X and Hi-C. A combination of multiple technologies is reported to improve the quality and completeness of genome assembly (Wang et al., 2023). An average of 27,767,526; 2,331,456; and 168,098,715 reads were accessed in 10x Genomics, Nanopore, and Hi-C libraries, respectively after pre-processing and quality check. **Supplementary Table S1** shows the detailed read statistics in different replicates and their average length in all three libraries. GC% was 39 for 10x Genomics and Hi-C read libraries, while Nanopore reads had 35% GC content.

*De-novo* genome assembly was generated using 10x Genomics libraries followed by mapping of Nanopore libraries onto *de-novo* genome assembly for further scaffolding. The nanopore raw read size ranged from 1000 bp to 222917 bp, with N50 (minimum length representing half of the total length of the assembly) as 26.08 Kb and 15.29 Mb for raw reads and scaffolds, respectively. Then, reads from HiC libraries were used for chromosome-level scaffolding, which is considered as the best choice for capturing the longest range DNA connectedness (Wang et al., 2023).

The genome assembly of *M. balsamina* and its assessment was found to have 3,710 scaffolds of 384,902,967 bp length and N50 of 30,984,295 bp (**Table 1**). BUSCO analysis, which uses universal single-copy orthologs, is considered as high-resolution quantifications of genomes, which facilitate informative comparisons and provides suggestions for improvements to assemblies or annotations (Simao et al., 2015). Assessment of this generated assembly shows 2,266 (97.4%) of 2,326 BUSCO to be complete and single copy (**Table 1**). The comparative statistics of *M. balsamina* assembly with other assemblies of related species showed the assembly size to be comparable with others while the N50 value (30.96 mb) was much improved than other assemblies (**Table 2**).

**Table 1:** *M. balsamina* assembly statistics.

Assembly parameters	Statistics
# contigs	3,710
# contigs (≥ 1000 bp)	3,702
# contigs (≥ 10000 bp)	569
# contigs (≥ 100000 bp)	67
# contigs (≥ 1000000 bp)	11
Largest contig (bp)	40,892,414
Average length (bp)	103,747
Smallest contig (bp)	957
Total length (bp)	384,902,967
Total length (≥ 1000 bp)	384,902,967
Total length (≥ 10000 bp)	384,902,967
Total length (≥ 100000 bp)	353,358,081
Total length (≥ 1000000 bp)	353,358,081
N50 (bp)	30,984,295
N75 (bp)	27,371,744
L50	6
L75	9
Total GC Content	35.43%
BUSCO	
Complete BUSCOs (C)	97.4%
Complete and single-copy BUSCOs (S)	95.3%
Complete and duplicated BUSCOs (D)	2.1%
Fragmented BUSCOs (F)	0.7%
Missing BUSCOs (M)	1.9%
Total BUSCO groups searched (*n*)	2326
Noncoding RNA	
tRNA	1,823
rRNA (large + small subunit)	270
sRNA	1
miRNA	150
spliceosomal RNA	129
snoRNA	961
Antisense RNA	15
SRP RNA	27

**Table 2:** Comparative statistics of *M.balsamina* genome assembly with genome assemblies of related species.

Assembly statistics	*Momordica. balsamina* (Current study)	Bitter gourd (OHB3-1)	Bitter gourd (Dali-1)	Bitter gourd (long read assembly)	Cucumber	Musk melon	Water melon	Bottle gourd
Genome size (Mb)	384.9	285.5	293.6	302.9	243.5	375	353.5	313.4
Chromosomes (Mb)	349.27	172.0	251.3	291	177.3	316.3	330.0	308.1
Unknown scaffolds (Mb)	35.63	60.2	85.5	96.27	72.8	87.5	93.5	98.3
N50 (Mb)	30.96	1.1	3.3	25	1.1	4.7	2.4	8.7
GC content (%)	35.4	36.4	35.4	–	32.2	33.2	32.8	–
Predicted genes	41652	45859	26427	–	26682	27427	23440	22472
Masked (%)	56.73	34.7	41.5	52.52	20.8	35.4	39.8	46.9
LTR content (%)	26.82	27.4	31.8	23.97	11.5	25.0	30.5	39.8

### 3.2 Annotation of genome assembly

Genome annotation is crucial to facilitate the utilization of assembled genomes in genetic studies. In the current study, homology-based inference, *in-silico* prediction techniques and merged transcriptomics data (of *Momordica charantia*) are merged into a single concordant annotation (Yandell and Ence, 2012). Genome annotation was done to identify TEs, ncRNA encoding genes, tRNAs, ncRNAs, SSR makers, TF binding sites and protein-encoding genes in the assembled genome.

**Figure 2:**
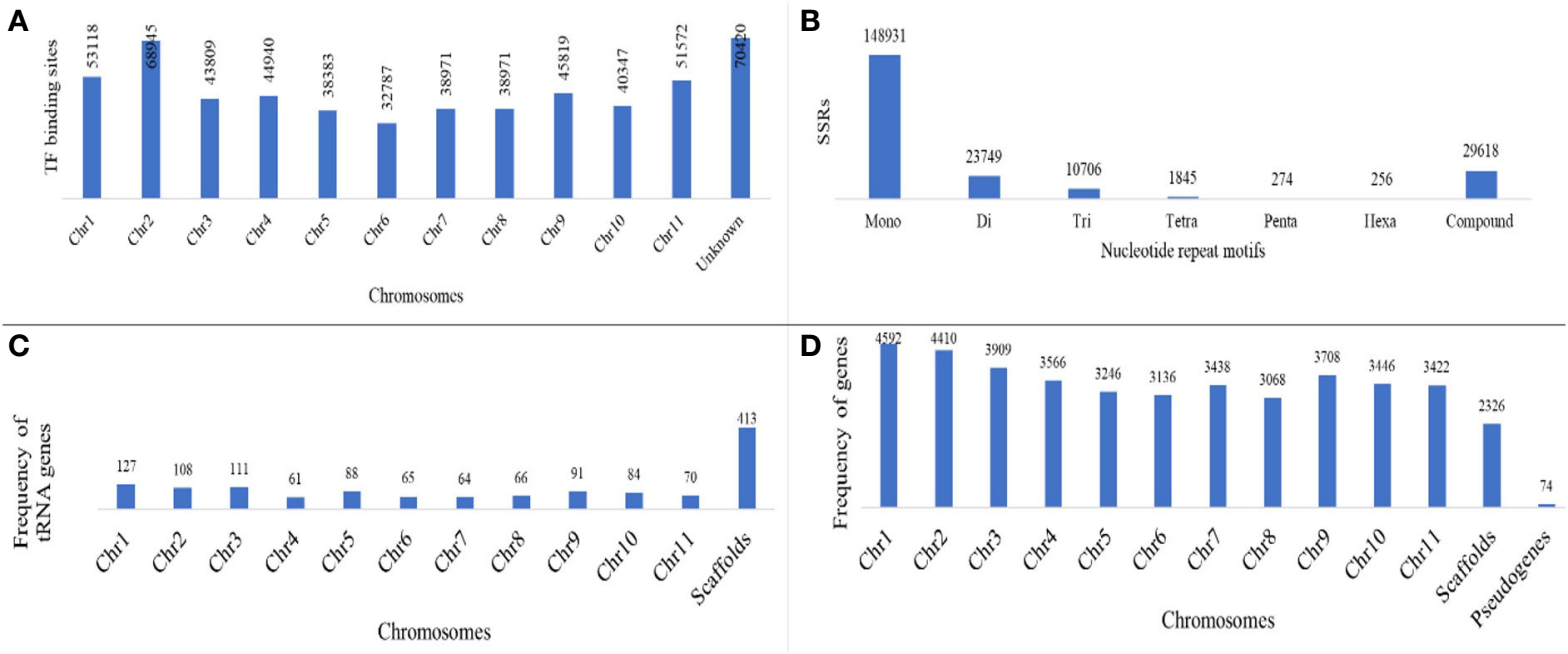
**(A)** Distribution of TF binding sites on different chromosomes; **(B)** frequency of SSRs of different nucleotide repeat motifs; **(C)** frequency of predicted tRNA genes and **(D)** protein coding genes distributed over chromosomes along with pseudogenes predicted in *M. balsamina* assembly.

Out of the total 384,902,967 bp length of 3,710 scaffolds of the assembled genome, 218,862,155 (56.73%) bases were masked. Frequencies of various classes of predicted TEs in genome assembly are delineated in **Table 3**. A significant proportion of TE class belonged to LTR elements, while 22.29% were found to be unclassified. The frequency of SINEs was the least (0.05%), while it was 3.02% for SINEs. A sum of 567,483 TF binding sites were predicted in *M. balsamina* genome and **Figure 2A** is showing chromosome wide distribution of TF binding sites. Maximum number of TF binding sites were observed in chromosome number 2 (~12%), followed by chromosome number 1 (~9%) and chromosome number 11 (~9%). Almost ~12% of TF binding sites were associated with the remaining unknown scaffolds (**Figure 2A**).

**Table 3:** Frequencies and proportion of various classes of TEs predicted in *M. balsamina* assembly.

Classes of TEs	Frequency	Length (bp)	Proportion
SINEs	746	181264	0.05%
LINEs	29,398	11,643,197	3.02%
LTR elements	101,274	103,473,791	26.82%
DNA transposons	18,021	9,880,729	2.56%
Simple repeat	125,651	4,585,002	1.19%
Low complexity	26,644	1,304,816	0.34%
Unclassified	322,593	85,978,857	22.29%

A total of 2,15,379 SSR markers were mined from the assembled genome. The highest number of SSR belonged to motif type mono-nucleotide (~69%), followed by di (~13%) and tri (~6%). A total of 29,618 (~9%) SSRs were compound type (**Figure 2B**). A total 3,376 different non-coding RNA genes were predicted in *M. balsamina* assembly, out of which 1,823 tRNA, 270 rRNA, 150 microRNA, 961 snoRNA, 27 SRP RNA, and 129 spliceosomal RNA genes were predicted (**Table 1**). Out of the total 1,823 predicted tRNA genes in *M. balsamina* assembly, their frequency distribution over chromosome 1 was highest, followed by chromosomes 3 and 2. A minimum number of tRNA genes were observed in chromosome 4 (**Figure 2C**). Apart from the chromosomes, higher number of tRNA genes were found localized on unknown scaffolds. **Figure 2D** shows the frequencies of protein-coding genes distributed over various chromosomes along with 74 pseudogenes predicted in *M. balsamina* assembly. It was observed that a higher number of protein-coding genes were found on chromosomes 1 (4,592), followed by chromosome 2 (4,410) and 3 (3,909).

### 3.3 Functional annotation of protein-coding genes

**Figure 3:**
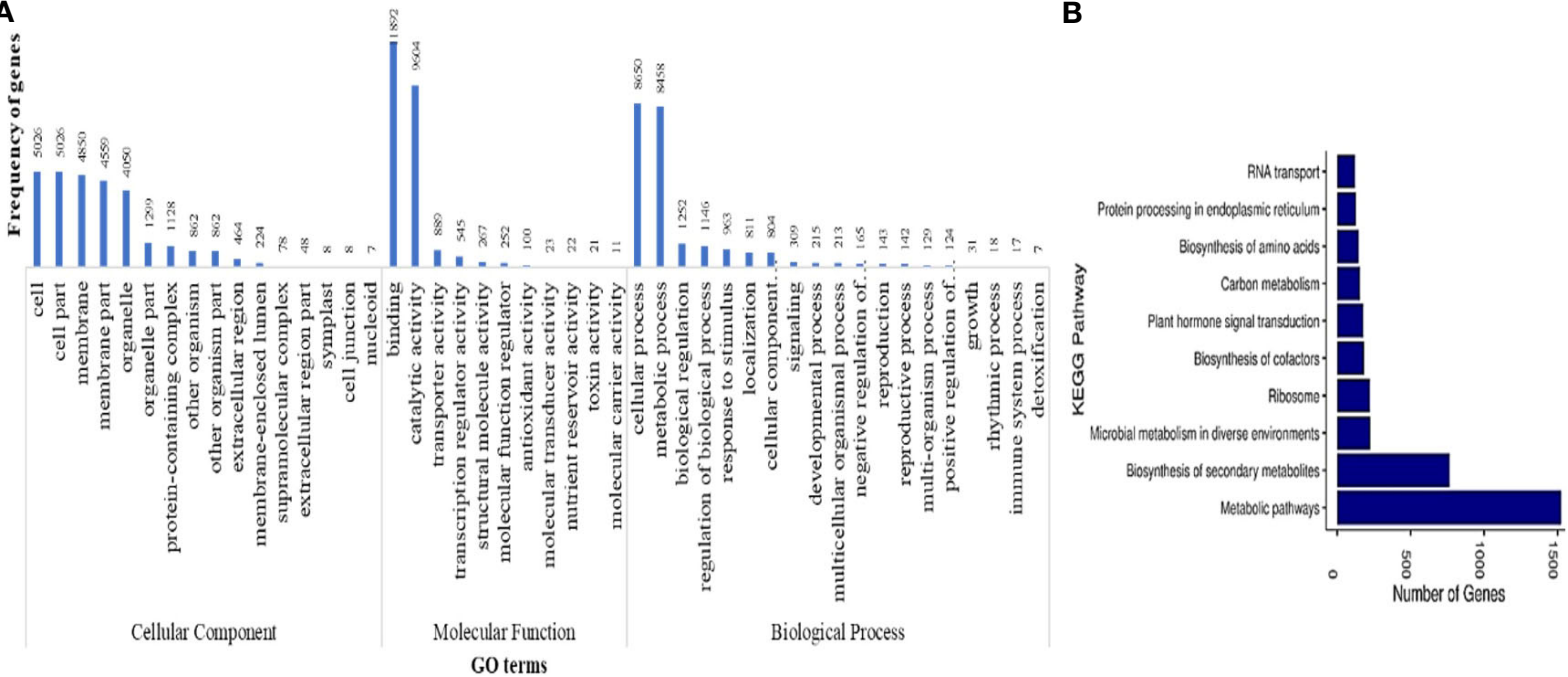
**(A)** GO terms associated with predicted protein coding genes and **(B)** top 10 KEGG pathways associated with annotated protein coding genes in *M. balsamina* assembly.

Functional annotation of protein-coding genes yielded a total of 33,450 genes that were annotated with NR database. GO analysis of these annotated genes showed 52 GO terms to be associated with 20,525 genes, of which 16, 12, and 25 were from cellular component, molecular function, and biological process classes, respectively. The GO terms were categorized into three classes, namely, molecular function, biological functions and cellular components. **Figure 3A** shows the GO terms associated with more than five protein-coding genes predicted in *M. balsamina* assembly. It was found that the GO terms named binding activities (11,892) followed by the catalytic activities (9,604) and transporter activities (889) were associated with most genes in molecular function class. In biological processes, cellular processes GO term (8,650) was the most frequent in genes, followed by metabolic processes (8,458) and biological regulations (1,252). Cell (5,026), cell part (5,026), and membrane (4,850) GO terms were the most frequent in cellular component class (**Figure 3A**). **Figure 3B** shows the top 10 KEGG pathways associated with 3,414 annotated genes in *M. balsamina* assembly. It was found that metabolic pathways (>1,500 genes involved) were the most abundant pathway, followed by biosynthesis of secondary metabolites (~700 genes involved) and microbial metabolism (~250 genes involved) in diverse environments.

### 3.4 Genes related to plant defence, medicinal properties, and sex expression

*M. balsamina* is well-known for its biotic and abiotic stress tolerance and medicinal properties. In the *M. balsamina* assembly, a total of 4,347 important disease resistance genes (R genes) were identified, out of which 1,174 genes encoded for nucleotide-binding site–leucine-rich repeat (NBS-LRR) domains along with 858 *RLP* and 273 *RLK* encoding genes, which are well known in resistance response in plants. In our study, we identified 67 heat stress-related genes, including a total of 34 heat stress factor genes (HSFs), contribute to thermo-tolerance through the regulation of heat shock proteins (HSPs). In addition, 29 *HSP* genes, predominantly encoding HSP70, and 17 small heat proteins (HSP20) were identified in the *M. balsamina* assembly. Similarly, 15 genes encoding proteins related to salinity tolerance in the *M. balsamina* assembly, including alkaline ceramidase (ACER), S-acyltransferase, salt stress root protein RS1-like, and protein RICE SALT SENSITIVE 3 isoforms were identified. Cucurbit crops are considered as models for deciphering the mechanism of sex determination in monoecious plant species and ethylene is considered to be the core regulator. To shed more light on this, in the current study, 06 genes related to ethylene biosynthesis were extracted. *M. balsamina* contains a diverse array of Cucurbitacin terpenoid compounds exhibiting anti-septic, anti-microbial, anti-bacterial, anti-viral (including anti-HIV), anti-inflammatory, anti-plasmodial, antioxidant, and analgesic properties (Thakur et al., 2009; Ramalhete et al., 2022). The genes related to terpenoid biosynthesis were searched in the genome to elucidate the mechanism behind the medicinal property exhibited by this species. Thirty-seven antioxidant activity related and 229 genes related to the biosynthesis of cucurbitacin, the key factors behind medicinal attributes of the *M. balsamina*, were detected. **Table 4** shows the frequencies of genes extracted with provided functions. GO terms of pathogenesis-related genes, heat tolerant genes, salt tolerance-related genes, sex determination-related genes, triterpenoid-related genes, cucurbitin-related genes, nutrition-related genes, and phloem-related genes are graphically represented in **Supplementary Figure S1**.

**Table 4:** Frequencies of genes associated with disease resistance, defence, salt stress, heat stress, sex determination, and secondary metabolite synthesis identified in *M. balsamina* assembly.

Function	Class	Description	Genes
Disease resistance	C	Coiled-coil domain	01
	CK	Coiled-coil and Kinase domains	379
	CL	Coiled-coil and LRR domains	59
	CLK	Coiled-coil, LRR and Kinase domains	23
	CN	Coiled-coil and NBS domains	197
	CNK	Coiled-coil, NBS and Kinase domains	129
	CNL	Coiled-coil domain, a nucleotide binding site and a leucine-rich repeat (CC-NB-LRR)	107
	CNT	Coiled-coil, NBS and TIR domains	02
	CT	Coiled-coil and TIR domains	24
	CTL	Coiled-coil, TIR, LRR domains	06
	CTNL	Coiled-coil, TIR, NBS and LRR domains	16
	KIN	Kinase domain	1376
	L	LRR domain	32
	N	NBS domain only, lack of LRR	388
	NL	NBS domain at N-terminal and LRR st the C-terminal, and lack of the CC domain	408
	RLK	Kinase domain, and an extracellular leucine-rich repeat (Kin-LRR)	273
	RLP	Receptor-like protein, groups those with a receptor serine-threonine kinase-like domain, and an extracellular leucine- rich repeat (ser/thr-LRR)	858
	T	TIR domain only, lack of LRR or NBS	46
	TN	TIR and NBS domains	12
	TNL	Toll-interleukin receptor-like domain, a nucleotide binding site and a leucine-rich repeat (TIR-NB-LRR)	62
	TRAN	Transmembrane helix domain	17
	Others	–	06
Heat stress tolerance	–	Hsp20, hsp70, hsfb1, hsfa2, hsfa4, hsfb4, hsf-a6	67
Salt stress tolerance	–	Alkaline ceramidase (ACER), S-acyltransferase, Salt stress root protein RS1-like, Protein RICE SALT SENSITIVE 3 isoforms	15
Sex determination	–	ACS (1-aminocyclopropane-1-carboxylate synthase)-1, ACS-7, ACS-CMA101, ACS-CMW-33	06
Secondary metabolite sysnthesis	Carotenoids (Nutrition)	Chloroplast specific lycopene beta cyclase, Phytoene destaurase/phytoene dehydrogenase, Prolycopene isomerase, Zeta-carotene desaturase, Lycopene epsilon cyclase.	05
	Cucurbitacins (Defence)	Oxidosqualene cyclase (OSC), Cytochrome P450 (CYP), Acetyltransferase (ACT), UDP-glucosyltransferase (UGT)	229
	Trierpenoids (Medicinal use)	Balsaminol, Balsaminoside, Balsaminagenin, Karavilagenin, Cucurbalsaminol, Balsaminapentaol, Megastigmane-type nor-isoprenoid, Pimarane-type diterpenes	19
Antioxidant activity	Abiotic stress tolerance	Glutatione S transferase (GST)	37

### 3.5 Orthologous genes, phylogenetic, and synteny analysis

**Figure 4:**
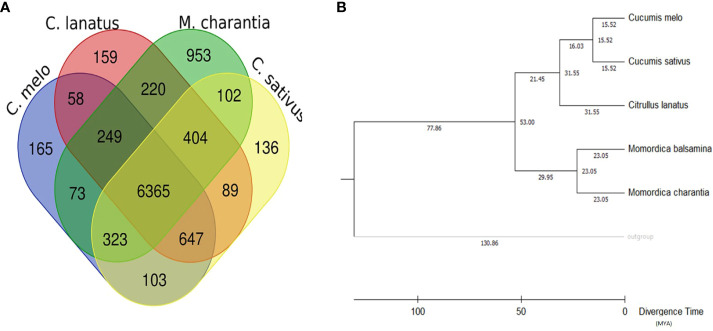
**(A)** Unique and overlapping *M. balsamina* genes found orthologous in other related species (*Cucumis sativus*, *Cucumis melo*, *Citrullus lanatus*, and *M. charantia*); **(B)** rooted phylogenetic tree represented in terms of divergence time (MYA: million years ago) based on whole genome assemblies of *M. balsamina* and other related species (*Cucumis sativus*, *Cucumis melo*, *Citrullus lanatus*, and *M. charantia*).

Comparative genetic parameters such as orthology, synteny, and phylogeny were utilized in the study to understand the genome composition, evolution and relatedness among the members of a family or clade at the nucleotide/molecular level. A total of 1,542 genes of *M. balsamina* were found orthologous with other related species considered in the present study. Frequencies of these genes are provided in **Table 5** along with the species with which these are found orthologous. The unique and overlapping *M. balsamina* genes found orthologous in other related species are delineated in **Figure 4A**. It is observed that 165, 159, 953, and136 *M. balsamina* genes were orthologous in *Cucumis melo*, *Citrullus lanatus*, *M. charantia*, and *Cucumis sativus*, respectively, only and the rest of the genes were orthologous in more than two species.

**Table 5:** Frequencies of *M. balsamina* orthologous genes and syntenic blocks found in other related species.

Species	*M. balsamina* orthologous genes	*M. balsamina s*yntenic blocks
*Momordica charantia*	8,845	306
*Citrullus lanatus*	8,308	264
*Cucumis sativus*	8,265	245
*Cucumis melo*	8,092	282

The syntenic relationship analyses of *M*. *balsamina* with other species were performed. In the synteny analysis, the sequences of related species were aligned, and conserved genes between the two genomes were identified as anchors, and then regions with more than seven anchors connecting two species were considered as synteny blocks. Frequencies of orthologous genes and syntenic blocks of *M. balsamina* with related species, *M. charantia*, *Citrullus lanatus*, *Cucumis sativus*, and *Cucumis melo* were found to be (8845, 306), (8308, 264), (8265, 245), and (8092, 282), respectively (**Table 5**). Also, the diagrammatic representation of syntenic blocks in the form of Circos figures is provided for synteny between *M. balsamina* and *Cucumis sativus*, *M. balsamina* and *Cucumis melo*, *M. balsamina* and *Citrullus lanatus*, *M. balsamina* and *M. charantia* (all scaffolds), and *M. balsamina* and *M. charantia* (scaffolds >100Mb), respectively (**Supplementary Figures S2A–E**). A general absence of a one-to-one relationship in the chromosomes between the *Momordica balsamina* and other cucurbit genomes was observed. However, syntenic loci of one chromosome of *Momordica balsamina* chromosome exhibited a syntenic relationship between one or two chromosomes of studied cucurbits. *Momoridica balsamina* Chr11 was syntenic to Chr6 and Chr7 of *Cucumis sativus* and Chr5 was syntenic to Chr3 and Chr4 of *C. sativus*. Similarly, Chr8 of *Momordica balsamina* was syntenic to Chr 11 of *C. melo.* Furthermore, Chr7 was colinear to Chr 2 and 12 of Melon. Synteny between *M. balsamina* Chr 7 and Chr2 of watermelon was observed. Furthermore, Chr 5 was syntenic to Chr5 and Chr7 of watermelon.

Maximum number of genes on each chromosome of *M. Balsamina* found homologous with genes on corresponding scaffolds of *M. charantia* are shown in **Supplementary Table 2**. In addition, the **Supplementary Figures S3A–K** show homologous genes on chromosomes 1–11 of *M. balsamina* with syntenic relationship with corresponding scaffolds *M. charantia*. The rooted phylogenetic tree was constructed to represent the phylogenetic relationship of *M. balsamina* with other related species, namely, *M. charantia*, *Cucumis melo*, *Cucumis sativus*, and *Citrullus lanatus* (**Figure 4B**). *M. balsamina* was observed to be more closely related to *M. charantia.*

### 3.6 Development of *M. balsamina* web-genomic resource

**Figure 5:**
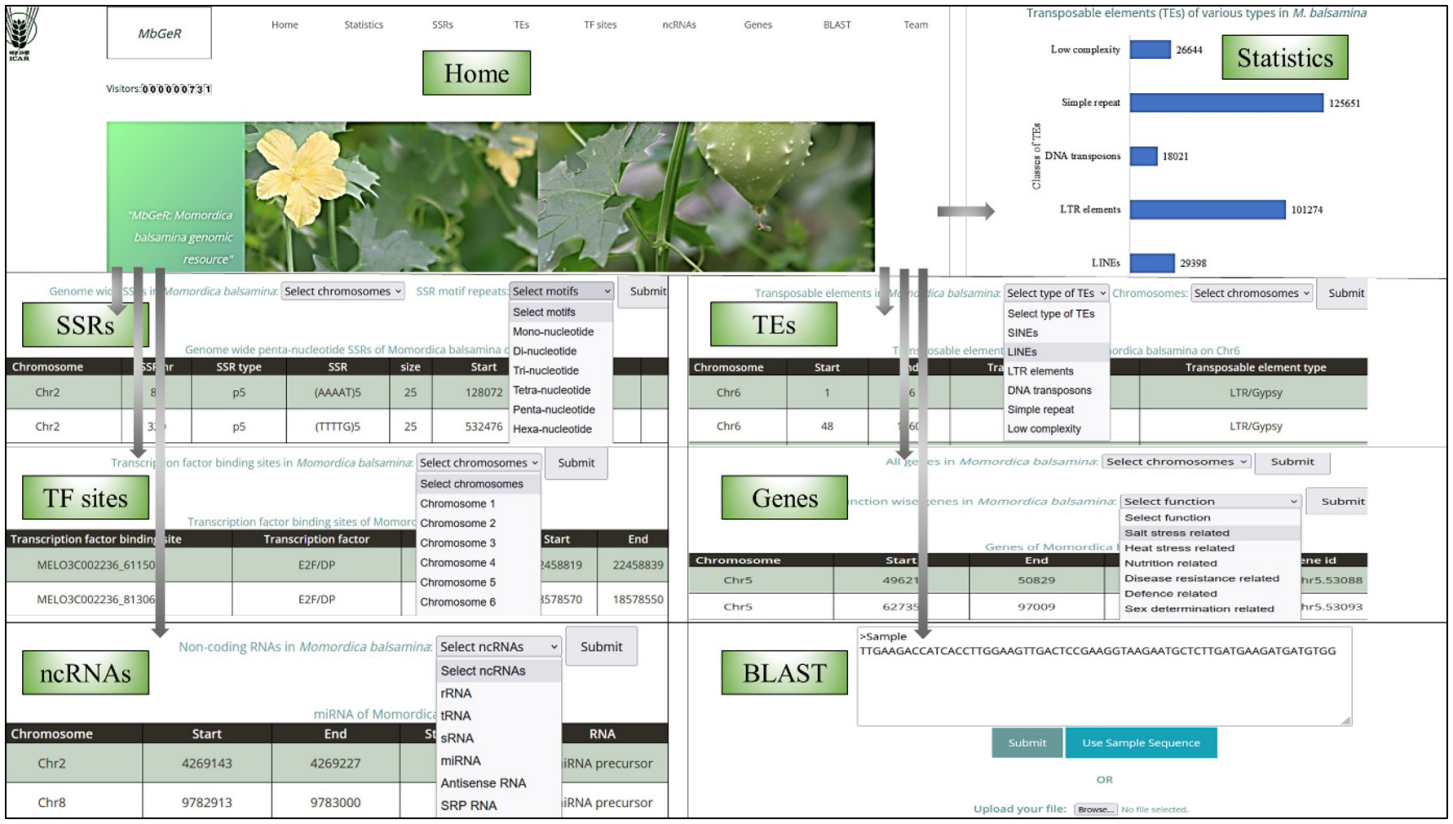
Various interfaces of *Momordica balsamina* web-genomic resource (*MbGeR*).

A web genomic resource for *M. balsamina*, named MbGeR, was developed from the output obtained after genomic data analyses of *M. balsamina* genome in the present study. Its web interface includes a home page with an introduction to MbGeR with horizontal and vertical tabs including statistics, SSRs, TEs, TF sites, ncRNAs, genes and team, each of which is linked to their respective pages (**Figure 5**). The statistics page provides summary statistics of data provided in genome resources in the form of histograms. Users are provided with flexible options to select SSR data on the desired 11 chromosomes of *M. balsamina* along with desired motifs on SSRs page. Users can choose TEs from the TEs page according to their desired types and chromosome numbers. TF sites provide options to choose TF binding sites on the desired chromosome. On the ncRNAs page, users can select non-coding RNAs among the various types. Gene’s page has two options: (i) selection of chromosomes for all genes extracted from the genome and (ii) choice of extracted genes associated with a certain function. Once the desired options are submitted on each of the mentioned the page, the output is displayed in tabular form in desired combinations of options. The Team page provides information and hyperlinked profiles of the team members involved in the study. The bitter gourd web resources, MbGeR is available for non-commercial use for research community at http://webtom.cabgrid.res.in/mbger/.

## 4 Discussion

CWRs are the primary source of diversity for utilization in crop improvement. Specifically, in crops with narrow genetic bases, the lack of diversity becomes the major bottlenecks in breeding program. To address the issue, close wild relatives inter-fertile with the cultivated crop species can be used as extended gene pool in crop improvement (Brozynska et al., 2016). CWRs evolve continuously in the natural environment and, hence, serve as a dynamic resource to access desirable genes to overcome several challenges in agriculture posed by increasing human population and climate change. Several workers have documented the wide-scale use of CWR to enhance agriculture production (Maxted et al., 2012; Fitzgerald, 2013; Dempewolf et al., 2014; Kell et al., 2015). It was estimated that about 30% of increased crop yields in the late 20th century can be attributed to the use of CWR in plant breeding programs (Pimentel et al., 1997). Hence, there is an increased need for the conservation and characterization of wild germplasm to utilize in crop improvement programmes. Molecular tools [e.g., simple sequence repeat (SSR) markers or microsatellites] were used in the past to characterize the CWR and to establish the relationship between wild and domesticated species (Dillon et al., 2007a; Sotowa et al., 2013). Recent DNA sequencing technology advancements increase opportunities to understand species at the whole-genome level (Edwards and Henry, 2011). Hence, genomic tools serve as the best strategy to characterize CWR and elucidate phylogenetic relationships between species, so that wild genetic diversity can be used in crop improvement (Kasem et al., 2010).

*M. balsamina*, Balsam apple is the closest wild species with cross-compatibility with *M. charantia*, exhibits greater tolerance to biotic stress, and possesses medicinal qualities (Rathode et al., 2021). Therefore, it is an ideal candidate species for harnessing natural variation within the primary gene pool and transferring desirable genes to cultivated *M. charantia*. Hence, genome characterization of this species proved vital for its usage in future breeding programs. In this study, we present the world’s first high-quality chromosome-level genome assembly of *M. balsamina*, with a genome size estimate of 384.90 Mb and an N50 of 30.96 Mb. This study used reads from multiple platforms (Oxford Nanopore, 10 X and Hi-C), which facilitates chromosome-level scaffolding with improved base accuracy. This assembly will facilitate targeted gene introgression between *M. balsamina* and *M. charantia*, enhancing tolerance and medicinal properties. Furthermore, this assembly, a combination of multiple technologies, can be used to improve further the quality and completeness of genome assembly of related species (Wang et al., 2023).

Approximately 89.44% (345 Mb) of the assembly was anchored on 11 chromosomes, while the remaining scaffolds remained un-localized. The quality of this assembly, based on the N50 value, surpasses that of previously published assemblies for other members of the *Cucurbitaceae* family, such as cucumber (Huang et al., 2009), melon (Garcia-Mas et al., 2012), watermelon (Guo et al., 2013), and bitter gourd (Cui et al., 2020 and Matsumura and Urasaki, 2020). Additionally, the BUSCO analysis revealed that the *M. balsamina* assembly contains 97.4% conserved core genes, a higher percentage compared to other *M. charantia* assemblies [M. cDali-11 (95.9%), TR (95.5%), and OHB3-1 (82.20%)] and related species: *C. lanatus* (86.50%), *C. melo* (86.9%), *Cucurbita pepo* (92.8%), *C. sativus* (94.8%), and *Lagenaria siceraria* (88.2%) assemblies (Cui et al., 2020). Gene space completeness as measured by single-copy standards, including universal single-copy orthologs (BUSCOs) and core gene families (CoreGFs) are widely used for evaluating genome assembly and annotation for its completeness and quality (Vaattovaara et al., 2019). Using estimates of gene content from hundreds of species and guided by evolution, BUSCO assessments provide comprehensible metrics to assess the completeness of genome and. hence it is considered high-resolution quantifications of the genomes (Simao et al., 2015). Therefore, with a high BUSCO score (97.4%), this assembly provides a comprehensive representation of the *M. balsamina* genome and serves as a valuable reference for studying the genome architecture and evolution of related cucurbits, including its closest cultivated species, *M. charantia*. The assembled genome of *M. balsamina* will aid in the identification of a greater number of genome-wide markers, allowing for the specific and accurate tracing of introgressed segments, which is crucial in interspecific introgression breeding, as reported by Qin et al. (2021). The assembly also revealed the presence of 632,098 TEs; 215,379 SSRs; 3,376 noncoding RNAs (ncRNAs); 567,483 TF binding sites; and 41,652 protein-coding genes. Many of these genes are associated with disease resistance (4421), heat stress tolerance (67), salt stress tolerance (15), carotenoid biosynthesis (05), cucurbitacin biosynthesis (229), terpenes related (19). antioxidant activity (37), and sex determination (06). Identifying these genes provides insights into the defence mechanisms, nutritional properties, and stress responses of *M. balsamina*.

TEs are well recognized for their role in genome evolution and regulation, providing alternative promoters, novel exons, neo-functionalization, and extensive rearrangements (Hoen & Bureau, 2015). A Comparison of our study’s assembly with recent studies on *M. charantia* assemblies by Cui et al., 2020, and Matsumura and Urasaki, 2020, revealed an improvement in the genome size of approximately 95 Mb and 84 Mb, respectively. This enhancement could be attributed to a higher repeat content in the *M. balsamina* genome than *M. charantia*. Our findings supported this hypothesis, as we observed that 56.73% (218.86 Mb) of the *M. balsamina* assembly was masked as TEs, which was higher than the percentages reported for *M. charantia* (52.52%), cucumber (20.8%), watermelon (39.8%), and muskmelon (35.4%) assemblies. LTR repeat content (26.82%) was the most abundant in *M. balsamina* genome. Higher LTR repeats are a common feature of cucurbit genomes evident from genomes of cucumber, melon, and watermelon (Huang et al., 2009; Garcia-Mas et al., 2012; Guo et al., 2013). In addition to this, the findings of the current experiment support the results of the previous studies on genome characterization of bitter gourd done by Urasaki et al., 2017 and Cui et al., 2020, which reported a higher accumulation of repeat content in the *Momordica* genus compared to *Cucumis* and *Citrullus*, particularly LTR repeats. However, LTR repeat content in the *M. balsamina* genome was less than in the Watermelon (30.5) and Bottle gourd (39.8). Earlier studies also speculated a differential rate of LTR retro transposon accumulation in the cucurbits as the reason behind the difference in the genome size among cucurbits. For instance, a higher accumulation of LTR retrotransposons is found in sponge gourd (Wu et al., 2020) and watermelon genome (Guo et al., 2013) than in cucumber (Huang et al., 2009). Hence, with absence of WGD (whole genome duplication) in cucurbits, TE might be playing vital role in genome expansion (Wu et al., 2020).

In our study, 3,376 noncoding RNA genes were annotated in the *M. balsamina* assembly. Extracted miRNAs, tRNAs, rRNAs, and other noncoding genes can be important resources for further studies. Additionally, we predicted 41,652 protein-coding genes in the *M. balsamina* assembly, a number comparable to the *M. charantia* OHB3-1 assembly (45859) by Urasaki et al. (2017), and significantly higher than the assemblies of *M. charantia* Dali-1 (26,427) by Cui et al., 2020, cucumber (26,682) by Huang et al. (2009), melon (27,427) by Garcia-Mas et al. (2012), and watermelon (23,440) by Guo et al. (2013). The variation in gene numbers could be attributed to the utilization of different transcript information during the annotation of genome assemblies or the loss of genetic diversity due to the domestication of cucurbits. Functional annotation of the protein-coding genes in our study revealed the presence of essential genes associated with detoxification, antioxidant activity, toxin activity, response to stimuli, immune system processes, defence, nutrient reservoir activity, and nutritional properties. These genes were also associated with pathways such as biosynthesis of secondary metabolites, plant hormone signal transduction, and protein processing in the endoplasmic reticulum.

*M. balsamina* is resistant to significant pest and diseases affecting cucurbits (Rathod et al., 2021). To understand the molecular basis for pest and pathogen resistance three major classes of *R*/resistance genes were searched in the genome. In the *M. balsamina* assembly, we identified 4,347 disease resistance genes (R genes), out of which 1,174 genes encoded NBS–LRR domains. These genes were grouped into two subfamilies based on the presence of either the toll/interleukin-1 receptor (TIR) domain or the coiled-coil (CC) domain at the N-terminal region, as described by Tameling et al. (2002). Additionally, we identified 858 *RLP* and 273 *RLK* encoding genes involved in conferring resistance response. These genes, such as *Cf* family proteins in tomatoes conferring resistance against *Cladosporium fulvum* fungus (Jones et al., 1994; Thomas et al., 1997) and *HcrVf2* in apples conferring apple scab resistance (Belfanti et al., 2004), were found in lower numbers compared to melon and cucumber. The number of R genes identified in *M. balsamina* was much higher than reported in bottle gourd, watermelon, cucumber, and melon. However, cucurbits generally have fewer NBS-LRR encoding genes than *Arabidopsis* (Baumgarten et al., 2003) and rice (Goff et al., 2002). Only 61 NBS containing resistance were found in the cucumber genome (Huang et al., 2009). Likewise, out of 411 genes associated with disease resistance in melon only 81 disease resistance genes encoded *NBS*, the *LRR* and the *TIR* domains (Garcia-Mas et al., 2012). Similarly, only 44 *NBS-LRR* genes were found in watermelon genome (Guo et al., 2013). So, in general, C*ucurrbitaceae* genomes possess comparatively a smaller number of R genes encoding *NBS-LRR* proteins (Lin et al., 2013). Hence, other mechanisms might be involved in stress response. For instance, in cucumber and *LOX* gene family expansion is speculated as the possible complementary mechanism to cope with pathogen invasion (Huang et al., 2009). However, in *M. balsamina*, it seems the defence mechanisms works through the involvement of “R” genes like the majority of crop plants. The variation in the number of *R* genes in cucurbits suggests that they are not conserved, and the differential expansion of NBS-encoding families could be attributed to segmental and whole-genome duplications during the evolution of plant species, as suggested by Wang et al. (2009). The higher number of *R* genes in the *M. balsamina* assembly suggests their potential use in improving resistance to a wide variety of prevalent biotic stresses in its closest relative, *M. charantia*.

In our study, we identified a total of 34 HSFs in the *M. balsamina* assembly, which was higher than the numbers reported for rice (25) by Chauhan et al. (2011), Arabidopsis (21) by Nover et al. (2001), and cucumber (23) by Chen et al. (2021). Among these genes, the primary heat stress factors identified were *HSFB1* (01), *HSFA2* (03), *HSFA4* (04), *HSFB4* (04), and *HSF-A6* (04), which contribute to thermo-tolerance through regulating HSPs as described by Ohama et al. (2017). Additionally, we identified 29 *HSP* genes, predominantly encoding HSP70, and 17 small heat proteins (HSP20) in the *M. balsamina* assembly. *HSP*s play an essential role in the regulation of HSFs and, subsequently, the expression of heat-responsive genes associated with heat tolerance. *HSP20* has been reported to contribute to heat stress tolerance in melon (Zheng et al., 2021), watermelon (He et al., 2019), cucumber (Chen et al., 2021), and pumpkin (Hu et al., 2021). Over-expression of *HSP70* has also been reported to significantly increase heat tolerance in watermelon, cabbage, and chilli (Park et al., 2013; Guo et al., 2015; Usman et al., 2015; Zhao et al., 2018; He et al., 2019). Therefore, the thermo-tolerance capacity of *M. balsamina* can be attributed to the identified important HSPs, which can be further functionally validated for future use. Similarly, we identified 15 genes encoding proteins related to salinity tolerance in the *M. balsamina* assembly, including ACER, S-acyltransferase, salt stress root protein RS1-like, and protein RICE SALT SENSITIVE 3 isoforms. These proteins have previously been reported to play a role in salinity tolerance in Arabidopsis by Wu et al. (2015) and in wheat by Kang et al. (2012). However, their role in salt tolerance in cucurbits has yet to be well documented. These identified genes with a possible role in salt tolerance can be further studied to understand the detailed physiological and molecular network associated with salt tolerance and improve the salt tolerance of related species through inter-specific introgression. Additionally, we found 37 glutathione S-transferase (GST) family genes in *M. balsamina*, which are vital antioxidant enzymes involved in reducing the damage caused by reactive oxygen species during abiotic stress (salt, drought, and cold) tolerance mechanisms (Venkateswarlu et al., 2012; Chan and Lam, 2014; Islam et al., 2019; and Song et al., 2021). GSTs are also involved in detoxification processes and protection against damage from various environmental factors (Dixon et al., 1998; (Esmaeili et al., 2009). The large number of identified GST family genes in *M. balsamina* suggests its high tolerance to abiotic stress, which can be harnessed to improve abiotic stress tolerance in *M. charantia*.

In the *M. balsamina* assembly, we identified five genes related to carotenoid biosynthesis, including chloroplast-specific lycopene beta-cyclase, phytoene desaturase/phytoene dehydrogenase, pro-lycopene isomerase, zeta-carotene desaturase, and lycopene epsilon cyclase. The overexpression of one or more carotenoid biosynthesis genes to produce carotene-rich varieties has been successfully employed in advanced vegetable improvement programs for crops such as tomatoes (Fraser et al., 2001), carrot (Fraser and Bramley, 2004), and potatoes (Diretto et al., 2007). Carotenoids contribute to color, serve as precursors of vitamin A, and have various health benefits, including reducing the risk of cancers and cardiovascular diseases (Paine et al., 2005; Aluru et al., 2008). Therefore, the transfer of these carotenoid biosynthesis genes from *M. balsamina* to *M. charantia* could be utilized to improve its nutritional value. Furthermore, we identified 229 genes related to cucurbitacin biosynthesis in the *M. balsamina* assembly. Cucurbitacins are signature bioactive compounds of the *Cucurbitaceae* family and confer a bitter taste to cucurbits (Chen et al., 2005). The identified genes encoding enzymes such as oxidosqualene cyclase, cytochromes P450, and acyltransferases are essential for cucurbitacin biosynthesis. Similar pathways and mechanisms are involved in the production of terpenoids across the genera of the *Cucurbitaceae* family (Huang et al., 2009; Shang et al., 2014). Moreover, we identified 19 genes related to the biosynthesis of other triterpenoids in the *M. balsamina* assembly. These triterpenoids have diverse medicinal properties, namely, anticancer, antidiabetic, anti-HIV, antimalarial, anti-inflammatory, and antimicrobial activities (Ramalhete et al., 2022). Many of these triterpenoids such as balsaminol, balsaminoside, balsaminagenin, karavilagenin, cucurbalsaminol, and balsaminapentaol (Ramalhete et al., 2009a; Ramalhete et al., 2009; Ramalhete et al., 2010; Ramalhete et al., 2011a; and Ramalhete et al., 2011b) have been previously isolated from *M. balsamina*, highlighting its potential as a source of bioactive compounds. These results confirm the value of *M. baslamina* in terms of its nutritional and therapeutic properties.

*M. balsamina* is a monoecious plant with separate male and female flowers on the same plant. Sex determination and expression in cucurbits have been extensively studied, and various phytohormones and their cross talk have been identified as key regulators (Chen et al., 2016; Wang et al., 2019). Ethylene, in particular, is considered a core regulator of sex expression in cucurbits (Yin and Quinn, 1995; Boualem et al., 2015; Chen et al., 2016). In the *M. balsamina* assembly, we identified six genes related to ethylene biosynthesis, including *ACS* (1-aminocyclopropane-1-carboxylate synthase) *ACS-7*, *ACS-CMA101*, and *ACS-CMW-33* genes. These genes are involved in the production of ethylene, which regulates sex expression in cucurbits. In *Cucumis sativus, ACS-1* is encoded by the *F* locus and is known to promote female sex expression by suppressing stamen development in bisexual flower primordial (Trebitsh et al., 1997; Mibus and Tatlioglu, 2004). Likewise, *ACS-7* is encoded by *A* locus (orthologue of the cucumber *M* gene) and is known to promote femaleness in monoecious melon lines, and a miss-sense mutation in *CmACS-7* led to andromonoecy, the predominant sex type of commercial melon (Boualem et al., 2008; Boualem et al., 2009). Similarly, two genes (*MOMC46*_189, *MOMC518*_1) encoding CmAcs-7 like protein and a gene (*MOMC3*_649) encoding CmACS 11 like protein were identified in *M. charantia* (Urasaki et al., 2017). *ACS* encoding genes for sex determination in *M. balsamina* and *M. charantia* were found orthologous by synteny analysis as well. This suggests the possible involvement ethylene regulated sex expression like all other cucurbits in *Momoridica* genus. The orthologous relationship of these *ACS* genes with those identified in *M. charantia* and other cucurbits suggests a highly conserved nature of sex-regulating genes across the *Cucurbitaceae* family. Additionally, our study revealed a high number of conserved genes (approximately 8,500) between *M. balsamina* and *M. charantia*, *Cucumis sativus*, *Cucumis melo*, and *Citrullus lanatus*, indicating a substantial level of genetic similarity and potential for comparative genomics studies among cucurbits.

Comparative plant genomics investigates the distinctiveness and differences among plant genomes. By comparing the genomes of closely and distantly related species, researchers can gain insights into the patterns and processes associated with plant genome evolution and identify functional regions within genomes (Caicedo and Purugganan, 2005). In this particular study, we conducted a genome comparison of *Momordica balsamina* with other related cucurbit species, namely, *Momordica charantia* (Bitter gourd), *Cucumis sativus* (Cucumber), *Cucumis melo* (Musk melon), and *Citrullus lanatus* (Watermelon), in order to identify syntenic and phylogenetic relationships. Our analysis revealed that *Momordica balsamina* shared the highest number of orthologous pairs (8,845) with *Momordica charantia*, followed by 8,265 orthologous pairs between *Momordica balsamina* and *Cucumis sativus*. Previous research by Garcia-Mas et al. (2012) identified 19,377 one-to-one ortholog pairs between *Cucumis melo* and *Cucumis sativus*.

Furthermore, we detected paralogous and orthologous relationships between the five studied *Cucurbitaceae* genomes, which can serve as a guide for translational research and facilitate the study of conserved economic traits. By utilizing conserved BUSCO genes (orthologous genes), we identified the evolutionary relationship between *Momordica balsamina*, *Momordica charantia*, *Citrullus lanatus*, *Cucumis sativus*, and *Cucumis melo*. Phylogenetic analysis done using *Vitis vinifera* as an outgroup classified *Momordica balsamina* and *Momordica charantia* to the same clade, indicating a close genetic relationship between these two species with a speciation/separation event estimated to have occurred 23 million years ago. Additionally, *Momordica* was found to be closer to *Citrullus* (Watermelon) than to *Cucumis*, suggesting a divergence around 53 million years ago. Previous studies by Urasaki et al. (2017); Jobst et al. (1998), and Schaefer et al. (2009) also reported a closer genetic association between bitter gourd and watermelon compared to cucumber or melon in phylogenetic and genetic analyses.

We performed synteny analysis to elucidate variations at the nucleotide level arising from mutations, duplications, chromosomal rearrangements, and gene family expansion or loss (Alkan et al., 2011). Synteny blocks, which identify regions of chromosomes shared between genomes that have a common order of homologous genes from a common ancestor, were identified to shed light on evolutionary relationships between species (Vergara and Chen, 2010). Previous synteny analysis in members of the *Cucurbitaceae* family helped to clarify the reason behind differences in basic chromosome number between *Cucumis sativus* and *C. melo* (Huang et al., 2009; Li et al., 2011). In our current study, we found the highest number of syntenic blocks between *Momordica balsamina* and *Momordica charantia* (306), followed by 282 syntenic blocks between *Momordica balsamina* and *Citrullus lanatus*, indicating a high level of synteny between *M. balsamina* and *M. charantia*, followed by watermelon (*Citrullus lanatus*). Previous synteny analyses also reported a high level of co-linearity between *Momordica* and *Citrullus* genomes (Urasaki et al., 2017; Cui et al., 2020). Our findings revealed a general absence of one-to-one relationships in the chromosomes between *Momordica balsamina* and other cucurbit genomes. This observation aligns with most of the synteny analyses conducted in cucurbits (Matsusmura and Urasaki, 2020); Wu et al., 2020, except for the study by Wu et al. (2017), which identified chromosome-level synteny between bottle gourd and melon (*C. melo*) and watermelon (*Citrullus lanatus*) genomes. The findings of our study, along with the synteny analysis by Matsusmura and Urasaki (2020), support the fact that most *Cucurbitaceae* genomes belong to a different clade than the genus *Momordica* (Renner and Schaefer, 2016). Therefore, the absence of one-to-one chromosome synteny between *Momordica* (*balsamina* and *charantia*) and other cucurbits may be attributed to higher structural re-arrangement in chromosomes after speciation.

In addition to the genome comparison and synteny analysis, we identified 215,379 SSRs and 567,483 TF binding sites (TFBSs). These data were incorporated into a genomic web resource called MbGeR, developed to provide access to the data extracted during this study. Characterizing the *M. balsamina* genome contributes to our understanding of the available gene pool that can be utilized to improve *M. charantia* through advanced plant breeding techniques. Due to the significant therapeutic values, resilience to biotic and abiotic stress and nutritional value of *M. balsamina*, this study offers valuable insights and a high-quality assembly and annotation of its genome, thereby assisting in the development of high-yielding and resistant varieties of this promising vegetable crop.

## 5 Conclusion

*M. balsamina* is the closest wild species of *M. charantia*, with higher resilience to biotic and abiotic stresses and greater medicinal and nutritional qualities. The present study provides the first high-quality chromosome-level genome assembly of *M. balsamina* with size 384.90 Mb and N5030.96 Mb using sequence data from 10x Genomics, Nanopore, and Hi-C platforms. Annotation of the provided assembly identified 215,379 SSRs; 632,098 TEs; 567,483 TF binding sites; 3,376 noncoding RNAs (tRNA, miRNA, snoRNA, and so forth) genes, and 41,652 protein coding genes. A sum of 4,347 disease resistance, 67 heat stress–related, 15 salt stress related, 229 cucurbitacin related, 19 terpenes related, 37 antioxidant activity, 05 carotenoid related, and 06 sex determination related genes were identified in *M. balsamina* assembly. Because of stress tolerance and better therapeutic values, *M. balsamina* will serve as a potential genomic resource, and provided assembly will help to boost the targeted gene introgression between *M. balsamina* and *M. charantia* species in developing high-yielding climate-smart and stress-resilient crop varieties. In addition, this high-quality genome assembly done using reads from multiple sequencing platforms can be used to improve further the quality and completeness of genome assembly of related species. The SSR markers obtained in this study would assist in linkage mapping, QTL and gene discovery, population genetics, evolutionary studies and gene regulation. The provided assembly will also help in identifying a higher number of genome-wide markers with greater specificity and accuracy to trace the introgressed segments during advanced breeding programs to improve resistance and medicinal values to high-yielding *M. charantia* varieties, which is significantly lost due to domestication of bitter gourd. Furthermore, the finding of comparative genome analysis (phylogeny and synteny) will be helpful to get insights into the patterns and processes associated with genome evolution and to uncover functional regions of cucurbit genomes.

## Data availability statement

Whole genome sequencing of Momordica balsamina: BioProject: PRJNA877043: First and high-quality assembly of Momordica balsamina, a potential genetic resource to improve tolerance and medicinal properties in bitter gourd, BioSample: SAMN30678163: Momordica balsamina genome; SRA: SRR21495983, SRR21495982 and SRR21495981. The assembly is submitted in NCBI with ID: SUB13995037.

## Author contributions

VN: Writing – original draft, Investigation. KS: Data curation, Writing – original draft. RE: Supervision, Writing – review & editing. VC: Writing – review & editing, Resources. SJ: Writing – review & editing, Data curation, Writing – original draft. MI: Data curation, Writing – review & editing. AM: Writing – review & editing, Resources, Supervision. HM: Supervision, Writing – review & editing. BG: Writing – review & editing, Investigation. GJ: Writing – review & editing. CK: Writing – review & editing, Supervision. AG: Writing – review & editing, Supervision. DK: Supervision, Writing – review & editing, Data curation, Investigation, Writing – original draft. SD: Supervision, Writing – review & editing, Data curation, Investigation, Writing – original draft. TB: Resources, Supervision, Writing – original draft, Writing – review & editing, Conceptualization.

## References

[B1] Ajji P. K. (2016). Functional characterization of a novel ribosome inactivating protein from Momordica balsamina (Deakin University, Australia: Doctoral dissertation, Deak in University). Available at: https://dro.deakin.edu.au/eserv/DU:30103049/ajji-functionalcharacterization-2017.pdf.

[B2] Ajji P. K. Binder M. J. Walder K. Puri M. (2017). Balsamin induces apoptosis in breast cancer cells via DNA fragmentation and cell cycle arrest. Mol. Cell. Biochem. 432 (1), 189–198. doi: 10.1007/s11010-017-3009-x 28378131

[B3] Alkan C. Coe B. P. Eichler E. E. (2011). Genome structural variation discovery and genotyping. Nat. Rev. Genet. 12 (5), 363–376. doi: 10.1038/nrg2958 21358748 PMC4108431

[B4] Aluru M. Xu Y. Guo R. Wang Z. Li S. White W.. (2008). Generation of transgenic maize with enhanced provitamin A content. J. Exp. Botany. 59 (13), 3551–3562. doi: 10.1093/jxb/ern212 18723758 PMC2561147

[B5] Andrews S. (2010) FASTQC. A quality control tool for high throughput sequence data. Available at: http://www.bioinformatics.babraham.ac.uk/projects/fastqc.

[B6] Aversano R. Contaldi F. Ercolano M. R. Grosso V. Iorizzo M. Tatino F.. (2015). The *Solanum commersonii* genome sequence provides insights into adaptation to stress conditions and genome evolution of wild potato relatives. Plant Cell. 27 (4), 954–968. doi: 10.1105/tpc.114.135954 25873387 PMC4558694

[B7] Baumgarten A. Cannon S. Spangler R. May G. (2003). Genome-level evolution of resistance genes in *Arabidopsis thaliana* . Genetics 165 (1), 309–319. doi: 10.1093/genetics/165.1.309 14504238 PMC1462749

[B8] Behera T. K. Behera S. Bharathi L. K. John K. J. Simon P. W. Staub J. E. (2010). Bitter gourd: botany, horticulture, breeding. Hortic. Rev. 37, 101–141. doi: 10.1002/9780470543672.ch2

[B9] Beier S. Thiel T. Münch T. Scholz U. Mascher M. (2017). MISA-web: a web server for microsatellite prediction. Bioinformatics 33 (16), 2583–2585. doi: 10.1093/bioinformatics/btx198 28398459 PMC5870701

[B10] Belaghzal H. Dekker J. Gibcus J. H. (2017). Hi-C 2.0: An optimized Hi-C procedure for high-resolution genome-wide mapping of chromosome conformation. Methods 123, 56–65. doi: 10.1016/j.ymeth.2017.04.004 28435001 PMC5522765

[B11] Belfanti E. Silfverberg-Dilworth E. Tartarini S. Patocchi A. Barbieri M. Zhu J.. (2004). The *HcrVf2* gene from a wild apple confers scab resistance to a transgenic cultivated variety. Proc. Natl. Acad. Sci. 101 (3), 886–890. doi: 10.1073/pnas.0304808101 14715897 PMC321776

[B12] Bharathi L. K. John K. J. (2013). Momordica Genus in Asia-An Overview Vol. p (New Delhi: Springer), 147). doi: 10.1007/978-81-322-1032-0

[B13] Bharathi L. K. Munshi A. D. Behera T. K. Vinod J. K.J. Das A. B. Bhat K. V.. (2012). Production and preliminary characterization of inter-specific hybrids derived from *Momordica* species. Curr. Sci. 103 (2), 178–186.

[B14] Bharathi L. K. Munshi A. D. Chandrashekaran S. Behera T. K. Das A. B. John K. J. (2011). Cytotaxonomical analysis of *Momordica* L. (Cucurbitaceae) species of Indian occurrence. J. Genet. 90 (1), 21–30. 21677385

[B15] Boualem A. Fergany M. Fernandez R. Troadec C. Martin A. Morin H.. (2008). A conserved mutation in an ethylene biosynthesis enzyme leads to andromonoecy in melons. Science 321 (5890), 836–838. doi: 10.1126/science.1159023 18687965

[B16] Boualem A. Troadec C. Camps C. Lemhemdi A. Morin H. Sari A.. (2015). A cucurbit androecy gene reveals how unisexual flowers develop and dioecy emerges. Science 350 (6261), 688–691. doi: 10.1126/science.aac8370 26542573

[B17] Boualem A. Troadec C. Kovalski I. Sari M. A. Perl-Treves R. Bendahmane A. (2009). A conserved ethylene biosynthesis enzyme leads to andromonoecy in two *Cucumis* species. PloS One 4 (7), e6144. doi: 10.1371/journal.pone.0006144 19578542 PMC2701604

[B18] Bowley S. R. Taylor N. L. (1987). “Introgressive hybridization,” in CRC handbook of plant science in agriculture, vol. 1 . Ed. Christie B. R. (Boca Raton: CRC Press), 23–59.

[B19] Brozynska M. Furtado A. Henry R. J. (2016). Genomics of crop wild relatives: expanding the gene pool for crop improvement. Plant Biotechnol. J. 14 (4), 1070–1085. doi: 10.1111/pbi.12454 26311018 PMC11389173

[B20] Caicedo A. L. Purugganan M. D. (2005). Comparative plant genomics. Frontiers and prospects. Plant Physiol. 138 (2), 545–547. doi: 10.1104/pp.104.900148 15955910 PMC1150366

[B21] Cao M. D. Nguyen S. H. Ganesamoorthy D. Elliott A. G. Cooper M. A. Coin L. J. (2017). Scaffolding and completing genome assemblies in real-time with nanopore sequencing. Nat. Commun. 8 (1), 1–10. doi: 10.1038/ncomms14515 28218240 PMC5321748

[B22] Chan C. Lam H. M. (2014). A putative lambda class glutathione S-transferase enhances plant survival under salinity stress. Plant Cell Physiol. 55 (3), 570–579. doi: 10.1093/pcp/pct201 24399237

[B23] Chan P. P. Lowe T. M. (2019). tRNAscan-SE: searching for tRNA genes in genomic sequences. Methods Mol. Biol. (Clifton NJ) 1962, 1–14. doi: 10.1007/978-1-4939-9173-0_1 PMC6768409 31020551

[B24] Chauhan H. Khurana N. Agarwal P. Khurana P. (2011). Heat shock factors in rice (*Oryza sativa* L.): genome-wide expression analysis during reproductive development and abiotic stress. Mol. Genet. Genomics 286 (2), 171–187. doi: 10.1007/s00438-011-0638-8 21792744

[B25] Chen J. C. Chiu M. H. Nie R. L. Cordell G. A. Qiu S. X. (2005). Cucurbitacins and cucurbitane glycosides: structures and biological activities. Natural product Rep. 22 (3), 386–399. doi: 10.1039/B418841C 16010347

[B26] Chen F. Dong W. Zhang J. Guo X. Chen J. Wang Z.. (2018). The sequenced angiosperm genomes and genome databases. Front. Plant Sci. 9. doi: 10.3389/fpls.2018.00418 PMC5909171 29706973

[B27] Chen F. Mackey A. J. Stoeckert C. J. Jr. Roos D. S. (2006). OrthoMCL-DB: querying a comprehensive multi-species collection of ortholog groups. Nucleic Acids Res. 34 (suppl_1), D363–D368. doi: 10.1093/nar/gkj123 16381887 PMC1347485

[B28] Chen H. Sun J. Li S. Cui Q. Zhang H. Xin F.. (2016). An ACC oxidase gene essential for cucumber carpel development. Mol. Plant 9 (9), 1315–1327. doi: 10.1016/j.molp.2016.06.018 27403533

[B29] Chen X. Wang Z. Tang R. Wang L. Chen C. Ren Z. (2021). Genome-wide identification and expression analysis of *Hsf* and *Hsp* gene families in cucumber (*Cucumis sativus* L.). Plant Growth Regul. 95 (2), 223–239. doi: 10.1007/s10725-021-00739-z

[B30] Conesa A. Gotz S. García-Gomez J. M. Terol J. Talón M. Robles M. (2005). Blast2GO: a universal tool for annotation, visualization and analysis in functional genomics research. Bioinformatics 21 (18), 3674–3676. doi: 10.1093/bioinformatics/bti610 16081474

[B31] Cui J. Yang Y. Luo S. Wang L. Huang R. Wen Q.. (2020). Whole-genome sequencing provides insights into the genetic diversity and domestication of bitter gourd (*Momordica* spp.). Horticulture Res. 7 (1), 85. doi: 10.1038/s41438-020-0305-5 PMC7261802 32528697

[B32] Dempewolf H. Baute G. Anderson J. Kilian B. Smith C. Guarino L. (2017). Past and future use of wild relatives in crop breeding. Crop Sci. 57 (3), 1070–1082. doi: 10.2135/cropsci2016.10.0885

[B33] Dempewolf H. Eastwood R. J. Guarino L. Khoury C. K. Müller J. V. Toll J. (2014). Adapting agriculture to climate change: a global initiative to collect, conserve, and use crop wild relatives. Agroecology Sustain. Food Syst. 38 (4), 369–377. doi: 10.1080/21683565.2013.870629

[B34] Dhillon N. P. Sanguansil S. Schafleitner R. Wang Y. W. McCreight J. D. (2016). Diversity among a wide Asian collection of bitter gourd landraces and their genetic relationships with commercial hybrid cultivars. J. Am. Soc. Hortic. Sci. 141 (5), 475–484. doi: 10.21273/JASHS03748-16

[B35] Dillon S. L. Lawrence P. K. Henry R. J. Price H. J. (2007). Sorghum resolved as a distinct genus based on combined ITS1, ndh F and Adh 1 analyses. Plant Systematics Evol. 268, 29–43. doi: 10.1007/s00606-007-0571-9

[B36] Diretto G. Al-Babili S. Tavazza R. Papacchioli V. Beyer P. Giuliano G. (2007). Metabolic engineering of potato carotenoid content through tuber-specific overexpression of a bacterial mini-pathway. PloS One 2 (4), e350. doi: 10.1371/journal.pone.0000350 17406674 PMC1831493

[B37] Dixon D. P. Cummins I. Cole D. J. Edwards R. (1998). Glutathione-mediated detoxification systems in plants. Curr. Opin. Plant Biol. 1 (3), 258–266. doi: 10.1016/S1369-5266(98)80114-3 10066594

[B38] Dudchenko O. Batra S. S. Omer A. D. Nyquist S. K. Hoeger M. Durand N. C.. (2017). *De novo* assembly of the *Aedes aEgypti* genome using Hi-C yields chromosome-length scaffolds. Science 356 (6333), 92–95. doi: 10.1126/science.aal3327 28336562 PMC5635820

[B39] Durand N. C. Shamim M. S. Machol I. Rao S. S. Huntley M. H. Lander E. S.. (2016). Juicer provides a one-click system for analysing loop-resolution Hi-C experiments. Cell Syst. 3 (1), 95–98. doi: 10.1016/j.cels.2016.07.002 27467249 PMC5846465

[B40] Edwards M. A. Henry R. J. (2011). DNA sequencing methods contributing to new directions in cereal research. J. Cereal Sci. 54 (3), 395–400. doi: 10.1016/j.jcs.2011.07.006

[B41] Erxleben A. Grüning B. (2020) Genome Annotation (Galaxy Training Materials). Available at: https://training.galaxyproject.org/training-material/topics/genome-annotation/tutorials/genome-annotation/tutorial (Accessed May 24 2022).

[B42] Esmaeili M. Shahrtash M. Moosavi F. Mohsenzadeh S. Mohabatkar H. (2009). Plant glutathione S-transferase function. Paper Presentation Proc. 6th Natl. Biotechnol. Congress Iran Tehran Iran.

[B43] Fitzgerald H. (2013).

[B44] Fraser P. D. Bramley P. M. (2004). The biosynthesis and nutritional uses of carotenoids. Prog. Lipid Res. 43 (3), 228–265. doi: 10.1016/j.plipres.2003.10.002 15003396

[B45] Flyman M. V. Afolayan A. J. (2007). Proximate and mineral composition of the leaves of Momordica balsamina L.: an under-utilized wild vegetable in Botswana. Int. J. Food Sci. Nutr. 58 (6), 419–423. doi: 10.1080/09637480701253417 17710585

[B46] Fraser P. D. Romer S. Kiano J. W. Shipton C. A. Mills P. B. Drake R.. (2001). Elevation of carotenoids in tomato by genetic manipulation. J. Sci. Food Agric. 81 (9), 822–827. doi: 10.1002/JSFA.908

[B47] García J. C. Guadagno A. Paytuvi-Gallart A. Saera-Vila A. Amoroso C. G. D’Esposito D.. (2021). PRGdb 4.0: an *updated database dedicated to genes involved in plant disease resistance process* . Nucleic Acids Res. 50 (D1), D1483–D1490. doi: 10.1093/nar/gkab1087 PMC8729912 34850118

[B48] Garcia-Mas J. Benjak A. Sanseverino W. Bourgeois M. Mir G. González V. M.. (2012). The genome of melon (*Cucumis melo* L.). Proc. Natl. Acad. Sci. 109 (29), 11872–11877. doi: 10.1073/pnas.1205415109 22753475 PMC3406823

[B49] Goff S. A. Ricke D. Lan T. H. Presting G. Wang R. Dunn M.. (2002). A draft sequence of the rice genome (*Oryza sativa* L. ssp. *japonica*). Science 296 (5565), 92–100. doi: 10.1126/science.1068275 11935018

[B50] Gramazio P. Yan H. Hasing T. Vilanova S. Prohens J. Bombarely A. (2019). Whole-genome resequencing of seven eggplant (*Solanum melongena*) and one wild relative (*S. incanum*) accessions provides new insights and breeding tools for eggplant enhancement. Front. Plant science. 1220. doi: 10.3389/fpls.2019.01220 PMC6791922 31649694

[B51] Guo M. Lu J. P. Zhai Y. F. Chai W. G. Gong Z. H. Lu M. H. (2015). Genome-wide analysis, expression profile of heat shock factor gene family (*CaHsfs*) and characterisation of *CaHsfA2* in pepper (*Capsicum annuum* L.). BMC Plant Biol. 15 (1), 151. doi: 10.1186/s12870-015-0512-7 26088319 PMC4472255

[B52] Guo S. Zhang J. Sun H. Salse J. Lucas W. J. Zhang H.. (2013). The draft genome of watermelon (*Citrullus lanatus*) and resequencing of 20 diverse accessions. Nat. Genet. 45 (1), 51–58. doi: 10.1038/ng.2470 23179023

[B53] Gurevich A. Saveliev V. Vyahhi N. Tesler G. (2013). QUAST: quality assessment tool for genome assemblies. Bioinformatics 29 (8), 1072–1075. doi: 10.1093/bioinformatics/btt086 23422339 PMC3624806

[B54] Hassan L. G. Umar K. J. (2006). Nutritional value of Balsam Apple (*Momordica balsamina* L.) leaves. Pakistan J. Nutr. 5 (6), 522–529. doi: 10.3923/pjn.2006.522.529

[B55] He Y. Fan M. Sun Y. Li L. (2019). Genome-wide analysis of watermelon *HSP20s* and their expression profiles and subcellular locations under stresses. Int. J. Mol. Sci. 20 (1), 12. doi: 10.3390/ijms20010012 PMC6337729 30577505

[B56] Hoen D. R. Bureau T. E. (2015). Discovery of novel genes derived from transposable elements using integrative genomic analysis. Mol. Biol. Evol. 32 (6), 1487–1506. doi: 10.1093/molbev/msv042 25713212

[B57] Hu Y. Zhang T. Liu Y. Li Y. Wang M. Zhu B.. (2021). Pumpkin (*Cucurbita moschata*) *HSP20* Gene Family Identification and Expression under Heat Stress. Front. Genet. 2062. doi: 10.3389/fgene.2021.753953 PMC8553033 34721541

[B58] Huang S. Li R. Zhang Z. Li L. I. Gu X. Fan W.. (2009). The genome of the cucumber, *Cucumis sativus* L. Nat. Genet. 41 (12), 1275–1281. doi: 10.1038/ng.475 19881527

[B59] Islam S. Sajib S. D. Jui Z. S. Arabia S. Islam T. Ghosh A. (2019). Genome-wide identification of glutathione S-transferase gene family in pepper, its classification, and expression profiling under different anatomical and environmental conditions. Sci. Rep. 9 (1), 1–15. doi: 10.1038/s41598-019-45320-x 31235811 PMC6591324

[B60] Jeffrey C. (1967). “Cucurbitaceae,” in Flora of tropical East Africa. Eds. Milne-Redhead C. E. Polhill R. M. (London, UK: Crown Agents for Overseas Governments and Administrations), 1–156.

[B61] Jin J. Tian F. Yang D. C. Meng Y. Q. Kong L. Luo J.. (2017). PlantTFDB 4.0: toward a central hub for transcription factors and regulatory interactions in plants. Nucleic Acids Res. 45, D1040–D1045. doi: 10.1093/nar/gkw982 27924042 PMC5210657

[B62] Jobst J. King K. Hemleben V. (1998). Molecular evolution of the internal transcribed spacers (ITS1 and ITS2) and phylogenetic relationships among the species of the family Cucurbitaceae. Mol. Phylo. Evol. 9, 204–219. doi: 10.1006/mpev.1997.0465 9562980

[B63] John K. J. (2005). Studies on ecogeography and genetic diversity of the genus Momordica L. @ in India (Kottayam, Kerala: Dissertation, Mahatma Gandhi University).

[B64] Jones D. A. Thomas C. M. Hammond-Kosack K. E. Balint-Kurti P. J. Jones J. D. (1994). Isolation of the tomato *Cf-9* gene for resistance to *Cladosporium fulvum* by transposon tagging. Science 266 (5186), 789–793. doi: 10.1126/science.7973631 7973631

[B65] Kang G. Li G. Zheng B. Han Q. Wang C. Zhu Y.. (2012). Proteomic analysis on salicylic acid-induced salt tolerance in common wheat seedlings (*Triticum aestivum* L.). Biochim. Biophys. Acta (BBA)-Proteins Proteomics. 1824 (12), 1324–1333. doi: 10.1016/j.bbapap.2012.07.012 22868037

[B66] Kasem S. Waters D. L. Rice N. Shapter F. M. Henry R. J. (2010). Whole grain morphology of Australian rice species. Plant Genet. Resour. 8 (1), 74–81. doi: 10.1017/S1479262109990189

[B67] Katoh K. Misawa K. Kuma K. I. Miyata T. (2002). MAFFT: a novel method for rapid multiple sequence alignment based on fast Fourier transform. Nucleic Acids Res. 30 (14), 3059–3066. doi: 10.1093/nar/gkf436 12136088 PMC135756

[B68] Kaur I. Yadav S. K. Hariprasad G. Gupta R. C. Srinivasan A. Batra J. K.. (2012). Balsamin, a novel ribosome-inactivating protein from the seeds of Balsam apple *Momordica balsamina* . Amino Acids 43 (2), 973–981. doi: 10.1007/s00726-011-1162-1 22120616

[B69] Kell S. Qin H. Chen B. Ford-Lloyd B. Wei W. Kang D.. (2015). China’s crop wild relatives: diversity for agriculture and food security. Agriculture Ecosyst. Environ. 209, 138–154. doi: 10.1016/j.agee.2015.02.012

[B70] Khare C. (2007). “*Momordica balsamina* Linn,” in Indian Medicinal Plants. Ed. Khare C. (New York, NY: Springer). doi: 10.1007/978-0-387-70638-2_1027

[B71] Krawinkel M. B. Ludwig C. Swai M. E. Yang R. Y. Chun K. P. Habicht S. D. (2018). Bitter gourd reduces elevated fasting plasma glucose levels in an intervention study among prediabetics in Tanzania. J. ethnopharmacology 216, 1–7. doi: 10.1016/j.jep.2018.01.016 29339109

[B72] Krzywinski M. Schein J. Birol I. Connors J. Gascoyne R. Horsman D.. (2009). Circos: an information aesthetic for comparative genomics. Genome Res. 19 (9), 1639–1645. doi: 10.1101/gr.092759.109 19541911 PMC2752132

[B73] Lewinski M. Bramkamp Y. Köster T. Staiger D. (2020). SEQing: web-based visualization of iCLIP and RNA-seq data in an interactive python framework. BMC Bioinf. 21 (1), 113. doi: 10.1186/s12859-020-3434-9 PMC7079501 32183735

[B74] Li D. Cuevas H. E. Yang L. Li Y. Garcia-Mas J. Zalapa J.. (2011). Syntenic relationships between cucumber (*Cucumis sativus* L.) and melon (C. melo L.) chromosomes as revealed by comparative genetic mapping. BMC Genomics 12 (1), 1–14. doi: 10.1186/1471-2164-12-396 PMC3199783 21816110

[B75] Limin F. Niu B. Zhu Z. Wu S. Li W. (2012). CD-HIT: accelerated for clustering the next generation sequencing data. Bioinformatics 28 (23), 3150–3152. doi: 10.1093/bioinformatics/bts565 23060610 PMC3516142

[B76] Lin X. Zhang Y. Kuang H. Chen J. (2013). Frequent loss of lineages and deficient duplications accounted for low copy number of disease resistance genes in cucurbitaceae. BMC Genomics 14, 1–13. 23682795 10.1186/1471-2164-14-335 PMC3679737

[B77] Lipman D. J. Pearson W. R. (1985). Rapid and sensitive protein similarity searches. Science 227 (4693), 1435–1441. doi: 10.1126/science.2983426 2983426

[B78] Mashiane P. Shoko T. Manhivi V. Slabbert R. Sultanbawa Y. Sivakumar D. (2022). A Comparison of bioactive metabolites, antinutrients, and bioactivities of african pumpkin leaves (*Momordica balsamina* L.) cooked by different culinary techniques. Molecules 27 (6), 1901. doi: 10.3390/molecules27061901 35335263 PMC8951283

[B79] Matsumura H. Urasaki N. (2020). “Genome sequence of bitter Gourd and Its Comparative Study with Other Cucurbitaceae Genomes,” in The Bitter Gourd Genome. Compendium of plant Genomes. Eds. Kole C. Matsumura H. Behera T. (Cham: Springer), 113–123). doi: 10.1007/978-3-030-15062-4_10

[B80] Maxted N. Kell S. Ford-Lloyd B. Dulloo E. Toledo Á. (2012). Toward the systematic conservation of global crop wild relative diversity. Crop Sci. 52 (2), 774–785. doi: 10.2135/cropsci2011.08.0415

[B81] Mibus H. Tatlioglu T. (2004). Molecular characterization and isolation of the *F/f* gene for femaleness in cucumber (*Cucumis sativus* L.). Theor. Appl. Genet. 109 (8), 1669–1676. doi: 10.1007/s00122-004-1793-7 15490106

[B82] Mishra K. C. Sahu P. R. Jha U. C. (1986). Balsam apple for your vegetable garden. Indian Horticulture J. 13.

[B83] Nawrocki E. P. Eddy S. R. (2013). Infernal 1.1: 100-fold faster RNA homology searches. Bioinformatics 29 (22), 2933–2935. doi: 10.1093/bioinformatics/btt509 24008419 PMC3810854

[B84] Nover L. Bharti K. Döring P. Mishra S. K. Ganguli A. Scharf K. D. (2001). Arabidopsis and the heat stress transcription factor world: how many heat stress transcription factors do we need? Cell Stress chaperones 6 (3), 177. doi: 10.1379/1466-1268(2001)006<0177:aathst>2.0.co;2 11599559 PMC434399

[B85] Ohama N. Sato H. Shinozaki K. Yamaguchi-Shinozaki K. (2017). Transcriptional regulatory network of plant heat stress response. Trends Plant Sci. 22 (1), 53–65. doi: 10.1016/j.tplants.2016.08.015 27666516

[B86] Paine J. A. Shipton C. A. Chaggar S. Howells R. M. Kennedy M. J. Vernon G.. (2005). Improving the nutritional value of Golden Rice through increased pro-vitamin A content. Nat. Biotechnol. 23 (4), 482–487. doi: 10.1038/nbt1082 15793573

[B87] Park H. J. Jung W. Y. Lee S. S. Song J. H. Kwon S. Y. Kim H.. (2013). Use of heat stress responsive gene expression levels for early selection of heat tolerant cabbage (*Brassica oleracea* L.). Int. J. Mol. Sci. 14 (6), 11871–11894. doi: 10.3390/ijms140611871 23736694 PMC3709761

[B88] Peter K. V. Abraham Z. (2007). Biodiversity in horticultural crops Vol. 1 (New Delhi, India: Daya Publisher).

[B89] Pimentel D. Wilson C. McCullum C. Huang R. Dwen P. Flack J.. (1997). Economic and environmental benefits of biodiversity. BioScience 47 (11), 747–757. doi: 10.2307/1313097

[B90] Qin X. Zhang Z. Lou Q. Xia L. Li J. Li M.. (2021). Chromosome-scale genome assembly of *Cucumis hystrix*—a wild species interspecifically cross-compatible with cultivated cucumber. Horticulture Res. 8 (1), 40. doi: 10.1038/s41438-021-00475-5 PMC7917098 33642577

[B91] Ramalhete C. da Cruz F. P. Lopes D. Mulhovo S. Rosario V. E. Prudêncio M.. (2011a). Triterpenoids as inhibitors of erythrocytic and liver stages of Plasmodium infections. Bioorganic medicinal Chem. 19 (24), 7474–7481. doi: 10.1016/j.bmc.2011.10.044 22071523

[B92] Ramalhete C. Gonçalves B. M. Barbosa F. Duarte N. Ferreira M. J. U. (2022). *Momordica balsamina*: phytochemistry and pharmacological potential of a gifted species. Phytochem. Rev. 21 (2), 617–646. doi: 10.1007/s11101-022-09802-7 35153639 PMC8821832

[B93] Ramalhete C. Lopes D. Molnár J. Mulhovo S. Rosário V. E. Ferreira M. J. U. (2011b). Karavilagenin C derivatives as antimalarial. Bioorganic medicinal Chem. 19 (1), 330–338. doi: 10.1016/j.bmc.2010.11.015 21129980

[B94] Ramalhete C. Lopes D. Mulhovo S. Molnar J. Rosário V. E. Ferreira M. J. U. (2010). New antimalarial with a triterpenic scaffold from *Momordica balsamina* . Bioorganic medicinal Chem. 18 (14), 5254–5260. doi: 10.1016/j.bmc.2010.05.054 20541427

[B95] Ramalhete C. Mansoor T. A. Mulhovo S. Molnár J. Ferreira M. J. U. (2009). Cucurbitane-type triterpenoids from the African plant *Momordica balsamina* . J. Natural products 72 (11), 2009–2013. doi: 10.1021/np900457u 19795842

[B96] Rathod V. Behera T. K. Munshi A. D. Gaikwad A. B. Singh S. Vinay N. D.. (2021). Developing partial interspecific hybrids of *Momordica charantia*× *Momordica balsamina* and their advance generations. Scientia Hortic. 281, 109985. doi: 10.1016/j.scienta.2021.109985

[B97] Renner S. S. Schaefer H. (2016). “Phylogeny and Evolution of the Cucurbitaceae,” in Genetics and Genomics of Cucurbitaceae. Plant Genetics and Genomics: Crops and Models, vol. 20 . Eds. Grumet R. Katzir N. Garcia-Mas J. (Cham: Springer). doi: 10.1007/7397_2016_14

[B98] Robinson R. W. Decker-Walters D. S. (1997). “Interspecific hybridization,” in Cucurbits. Eds. Robinson R. Decker-Walters D. S., (Oxon, U.K: CAB Intl.) 51–55. doi: 10.1073/pnas.81.24.8014

[B99] Robinson J. T. Turner D. Durand N. C. Thorvaldsdottir H. Mesirov J. P. Aiden E. L. (2018). Juicebox. js provides a cloud-based visualization system for Hi-C data. Cell Syst. 6 (2), 256–258. doi: 10.1016/j.cels.2018.01.001 29428417 PMC6047755

[B100] Saghai-Maroof M. A. Jorgensen R. A. Allard R. W. (1984). Ribosomal DNA spacer-length polymorphisms in barley: Mendelian inheritance, chromosomal location and population dynamics. Proc. Natl. Acad. Sci. U.S.A. 81, 8014–8018. 6096873 10.1073/pnas.81.24.8014 PMC392284

[B101] Sato S. Tabata S. Hirakawa H. Asamizu E. Shirasawa K. Isobe S.. (2012). The tomato genome sequence provides insights into fleshy fruit evolution. Nature 485 (7400), 635–641. doi: 10.1038/nature11119 22660326 PMC3378239

[B102] Schaefer H. Heibl C. Renner S. S. (2009). Gourds afloat: a dated phylogeny reveals an Asian origin of the gourd family (Cucurbitaceae) and numerous oversea dispersal events. Proc. R. Soc. B: Biol. Sci. 276 (1658), 843–851. doi: 10.1098/rspb.2008.1447 PMC2664369 19033142

[B103] Shang Y. Ma Y. Zhou Y. Zhang H. Duan L. Chen H.. (2014). Biosynthesis, regulation, and domestication of bitterness in cucumber. Science 346 (6213), 1084–1088. doi: 10.1126/science.1259215 25430763

[B104] Simao F. A. Waterhouse R. M. Ioannidis P. Kriventseva E. V. Zdobnov E. M. (2015). BUSCO: assessing genome assembly and annotation completeness with single-copy orthologs. Bioinformatics. 31 (19), 3210–3212. doi: 10.1093/bioinformatics/btv351 26059717

[B105] Singh A. K. (1990). “Cytogenetics and evolution in the cucurbitaceae,” in Biology and Utilization of Cucurbitaceae. Eds. Bates D. M. Robinson R. W. Jeffrey C. (Ithaca, New York, London: Comstock Publishing Associates, Cornell University Press), 10–28.

[B106] Singh B. P. (1991). Interspecific hybridization in between new and old-world species of *Luffa* and its phylogenetic implication. Cytologia 56 (3), 359–365. doi: 10.1508/cytologia.56.359

[B107] Soderlund C. Bomhoff M. Nelson W. M. (2011). SyMAP v3. 4: a turnkey synteny system with application to plant genomes. Nucleic Acids Res. 39 (10), e68–e68. doi: 10.1093/nar/gkr123 21398631 PMC3105427

[B108] Song W. Zhou F. Shan C. Zhang Q. Ning M. Liu X.. (2021). Identification of Glutathione S-Transferase Genes in Hami Melon (*Cucumis melo* var. *saccharinus*) and Their Expression Analysis under Cold Stress. Front. Plant Sci. 12. doi: 10.3389/fpls.2021.672017 PMC8217883 34168669

[B109] Sotowa M. Ootsuka K. Kobayashi Y. Hao Y. Tanaka K. Ichitani K.. (2013). Molecular relationships between Australian annual wild rice, Oryza meridionalis, and two related perennial forms. Rice 6, 1–19. doi: 10.1186/1939-8433-6-26 24280095 PMC3874672

[B110] Tameling W. I. Elzinga S. D. Darmin P. S. Vossen J. H. Takken F. L. Haring M. A.. (2002). The tomato R gene products I-2 and MI-1 are functional ATP binding proteins with ATPase activity. Plant Cell 14 (11), 2929–2939. doi: 10.1105/tpc.005793 12417711 PMC152737

[B111] Tan M. Ye J. Turner N. Hohnen-Behrens C. Ke C. Tang C.. (2008). Antidiabetic activities of triterpenoids isolated from bitter melon associated with activation of the AMPK pathway. Chem. Biol. 15 (3), 263–273. doi: 10.1016/j.chembiol.2008.01.013 18355726

[B112] Tang H. Krishnakuar V. Li J. (2015). jcvi: JCVI utility libraries. Zenodo. doi: 10.105281/zenodo31631

[B113] Thakur G. S. Bag M. Sanodiya B. S. Bhadauriya P. Debnath M. Prasad G. B. K. S.. (2009). *Momordica balsamina*: a medicinal and neutraceutical plant for health care management. Curr. Pharm. Biotechnol. 10 (7), 667–682. doi: 10.2174/138920109789542066 19751180

[B114] Thiel T. Michalek W. Varshney R. Graner A. (2003). Exploiting EST databases for the development and characterization of gene-derived SSR-markers in barley (*Hordeum vulgare* L.). Theor. Appl. Genet. 106, 411–422. doi: 10.1007/s00122-002-1031-0 12589540

[B115] Thomas C. M. Jones D. A. Parniske M. Harrison K. Balint-Kurti P. J. Hatzixanthis K.. (1997). Characterization of the tomato *Cf-4* gene for resistance to *Cladosporium fulvum* identifies sequences that determine recognition specificity in *Cf-4* and *Cf-9* . Plant Cell 9 (12), 2209–2224. doi: 10.1105/tpc.9.12.2209 9437864 PMC157069

[B116] Thompson J. D. Higgins D. G. Gibson T. J. (1994). CLUSTAL W: improving the sensitivity of progressive multiple sequence alignment through sequence weighting, position-specific gap penalties and weight matrix choice. Nucleic Acids Res. 22 (22), 4673–4680. doi: 10.1093/nar/22.22.4673 7984417 PMC308517

[B117] Trebitsh T. Staub J. E. O’Neill S. D. (1997). Identification of a 1-aminocyclopropane-1-carboxylic acid synthase gene linked to the female (*F*) locus that enhances female sex expression in cucumber. Plant Physiol. 113 (3), 987–995. doi: 10.1104/pp.113.3.987 9085580 PMC158220

[B118] TrimGalore (The Babraham Institute by @ FelixKrueger). Available at: https://www.bioinformatics.babraham.ac.uk/projects/trim_galore/.

[B119] Trivedi R. N. Roy R. P. (1972). Cytological studies in some species of Momordica. Genetica 43 (2), 282–291. doi: 10.1007/BF00123635

[B120] Untergasser A. Cutcutache I. Koressaar T. Ye J. Faircloth B. C. Remm M.. (2012). Primer3—new capabilities and interfaces. Nucleic Acids Res. 40 (15), e115–e115. doi: 10.1093/nar/gks596 22730293 PMC3424584

[B121] Urasaki N. Takagi H. Natsume S. Uemura A. Taniai N. Miyagi N.. (2017). Draft genome sequence of bitter gourd (*Momordica charantia*), a vegetable and medicinal plant in tropical and subtropical regions. DNA Res. 24 (1), 51–58. doi: 10.1093/dnares/dsw047 28028039 PMC5381343

[B122] Usman M. G. Rafii M. Y. Ismail M. R. Malek M. A. Latif M. A. (2015). Expression of target gene *Hsp70* and membrane stability determine heat tolerance in chili pepper. J. Am. Soc Hortic. Sci. 140 (2), 144–150. doi: 10.21273/JASHS.140.2.144

[B123] Vaattovaara A. Leppälä J. Salojärvi J. Wrzaczek M. (2019). High-throughput sequencing data and the impact of plant gene annotation quality. J. Exp. Bot. 70 (4), 1069–1076. 30590678 10.1093/jxb/ery434 PMC6382340

[B124] Venkateswarlu B. Shanker A. K. Shanker C. Maheswari M. (2012). Crop stress and its management: perspectives and strategies (DORDRECHT, Netherlands: Springer Science & Business Media). doi: 10.1007/978-94-007-2220-0

[B125] Vergara I. A. Chen N. (2010). Large synteny blocks revealed between *Caenorhabditis elegans* and *Caenorhabditis briggsae* genomes using OrthoCluster. BMC Genomics 11 (1), 1–13. doi: 10.1186/1471-2164-11-516 20868500 PMC2997010

[B126] Walker B. J. Abeel T. Shea T. Priest M. Abouelliel A. Sakthikumar S.. (2014). Pilon: an integrated tool for comprehensive microbial variant detection and genome assembly improvement. PloS One 9 (11), e112963. doi: 10.1371/journal.pone.0112963 25409509 PMC4237348

[B127] Wang R. Jin Q. Yao C. Zhong Y. Wu T. (2019). RNA-Seq analysis of gynoecious and weak female cucumber revealing the cell cycle pathway may regulate sex determination in cucumber. Gene 687, 289–297. doi: 10.1016/j.gene.2018.11.071 30471333

[B128] Wang X. Tang H. Bowers J. E. Paterson A. H. (2009). Comparative inference of illegitimate recombination between rice and sorghum duplicated genes produced by polyploidization. Genome Res. 19 (6), 1026–1032. doi: 10.1101/gr.087288.108 19372385 PMC2694483

[B129] Wang J. Veldsman W. P. Fang X. Huang Y. Xie X. Lyu A.. (2023). Benchmarking multi-platform sequencing technologies for human genome assembly. Briefings Bioinf. 24 (5), bbad300. doi: 10.1093/bib/bbad300 37594299

[B130] Weeden N. F. Robinson R. W. (1986). Allozyme segregation ratios in the interspecific cross *Cucurbita maxima* x *C. Ecuadorensis* suggest that hybrid breakdown is not caused by minor alterations in chromosome structure. Genetics 114 (2), 593–609. doi: 10.1093/genetics/114.2.593 17246350 PMC1202959

[B131] Wehner T. C. Naegele R. P. Myers J. R. Narinder P. S. Crosby K. (2020). Cucurbits. 2nd ed (Parlier, CA, USA: CABI). Available at: https://www.ars.usda.gov/research/publications/publication/?seqNo115=360003.

[B132] Weisenfeld N. I. Kumar V. Shah P. Church D. M. Jaffe D. B. (2017). Direct determination of diploid genome sequences. Genome Res. 27 (5), 757–767. 28381613 10.1101/gr.214874.116 PMC5411770

[B133] Wu S. Lau K. H. Cao Q. Hamilton J. P. Sun H. Zhou C.. (2018). Genome sequences of two diploid wild relatives of cultivated sweet potato reveal targets for genetic improvement. Nat. Commun. 9 (1), 1–12. doi: 10.1038/s41467-018-06983-8 30389915 PMC6214957

[B134] Wu X. Li J. Liu Z. Yin J. Chang Y. Rong C.. (2015). The Arabidopsis ceramidase *AtACER* functions in disease resistance and salt tolerance. Plant J. 81 (5), 767–780. doi: 10.1111/tpj.12769 25619405

[B135] Wu S. Shamimuzzaman M. D. Sun H. Salse J. Sui X. Wilder A.. (2017). The bottle gourd genome provides insights into Cucurbitaceae evolution and facilitates mapping of a Papaya ring-spot virus resistance locus. Plant J. 92 (5), 963–975. doi: 10.1111/tpj.13722 28940759

[B136] Wu H. Zhao G. Gong H. Li J. Luo C. He X.. (2020). A high-quality sponge gourd (*Luffa cylindrica*) genome. Horticulture Res. 7 (1), 128. doi: 10.1038/s41438-020-00350-9 PMC7395165 32821411

[B137] Yandell M. Ence D. (2012). A beginner’s guide to eukaryotic genome annotation. Nat. Rev. Genet. 13 (5), 329–342. doi: 10.1038/nrg3174 22510764

[B138] Yin T. Quinn J. A. (1995). Tests of a mechanistic model of one hormone regulating both sexes in *Cucumis sativus* (Cucurbitaceae). Am. J. Bot. 82 (12), 1537–1546. doi: 10.1002/j.1537-2197.1995.tb13856.x

[B139] Zhao Q. Chen W. Bian J. Xie H. Li Y. Xu C.. (2018). Proteomics and phosphoproteomics of heat stress-responsive mechanisms in spinach. Front. Plant Sci. 9. doi: 10.3389/fpls.2018.00800 PMC6029058 29997633

[B140] Zhao C. Qiu J. Agarwal G. Wang J. Ren X. Xia H.. (2017). Genome-Wide Discovery of Microsatellite Markers from Diploid Progenitor Species, *Arachis duranensis* and A. ipaensis, and Their Application in Cultivated Peanut (*A. hypogaea*). Front. Plant Science. 8. doi: 10.3389/fpls.2017.01209 PMC5513918 28769940

[B141] Zheng Y. Chen B. Zhi C. Qiao L. Liu C. Pan Y.. (2021). Genome-wide identification of small heat shock protein (*HSP20*) homologs in three cucurbit species and the expression profiles of *CsHSP20s* under several abiotic stresses. Int. J. Biol. Macromolecules 190, 827–836. doi: 10.1016/j.ijbiomac.2021.08.222 34492251

