# Supplementary material for: High-quality Momordica balsamina genome elucidates its potential use in improving stress resilience and therapeutic properties of bitter gourd
Source: Front Plant Sci. 2024 Jan 24;14:1258042. doi: 10.3389/fpls.2023.1258042 (PMC10851156; doi:10.3389/fpls.2023.1258042)
Supplement: Supplementary file 1 [file DataSheet_1.docx]

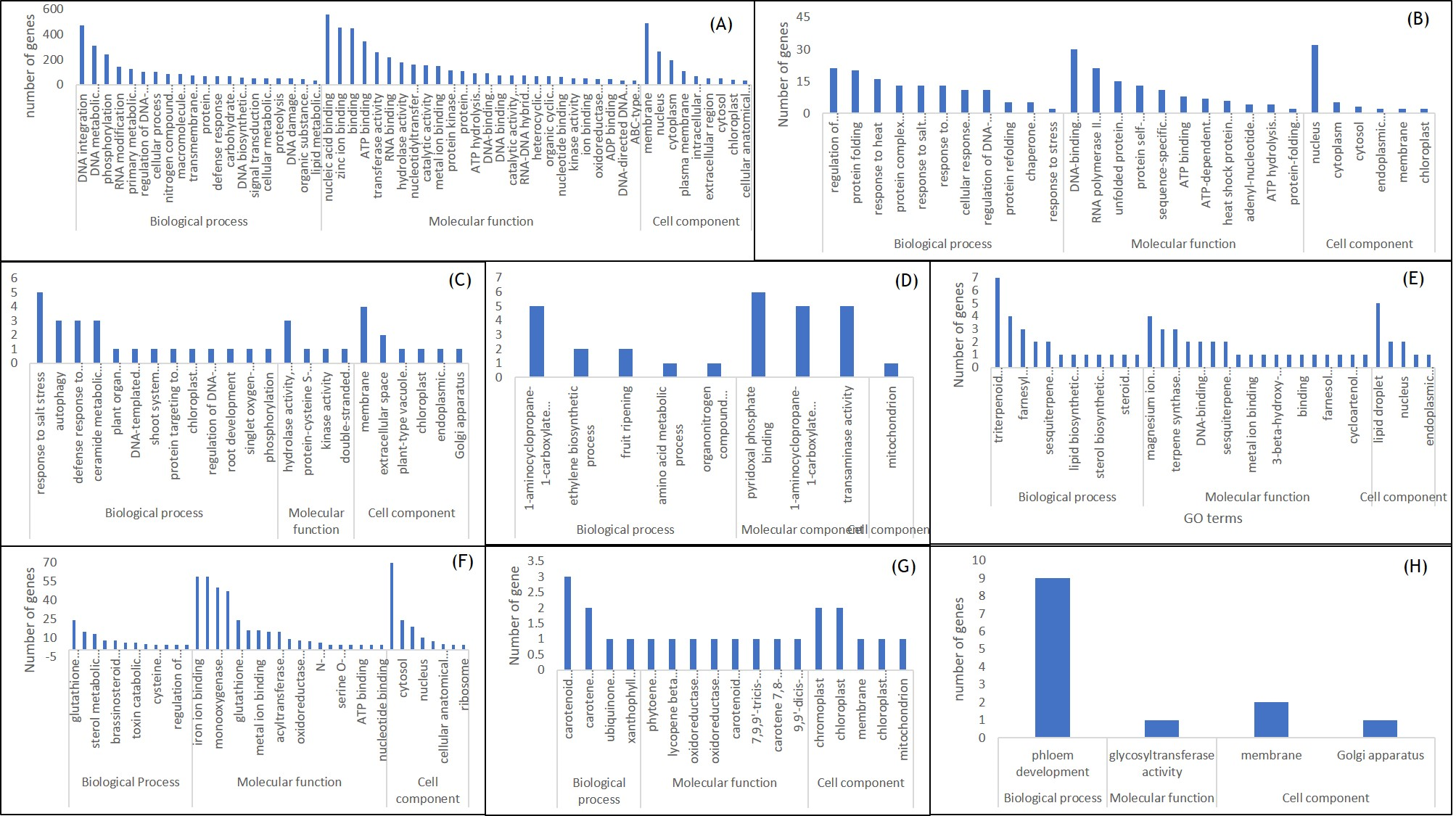


**Supplementary Figure 1**: Graphical representation of GO terms of (A) Pathogenesis related genes, (B) Heat tolerant genes, (C) Salt tolerance related genes, (D) Sex determination related genes and (E) triterpenoid related genes, (F) Cucurbitin related genes, (G) Nutrition related genes and (H) Phloem related genes


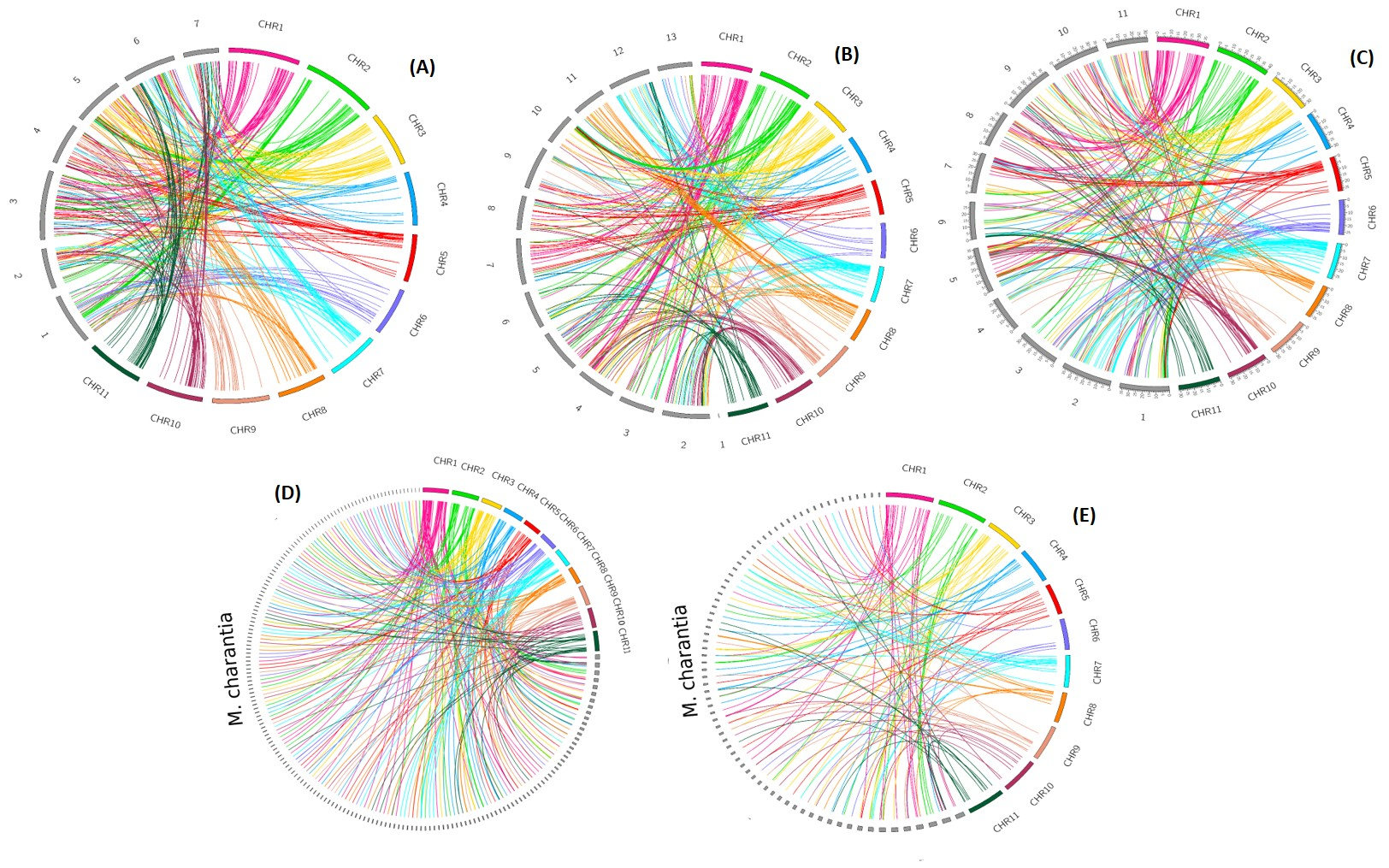


**Supplementary Figure 2**: Chromosome-wise variation mapping of *M. balsamina* with species, namely, (A) 7 chromosomes of *Cucumis sativus*, (B) 13 chromosomes of *Cucumis melo*, (C) 11 chromosomes of *Citrullus lanatus*, (D) all *M. charantia* scaffolds, and (E) scaffolds >100Mb of *M. charantia* scaffolds. The colored ribbons represent 11 chromosomes of *M. balsamina* and grey ribbons represent the chromosomes of compared species.


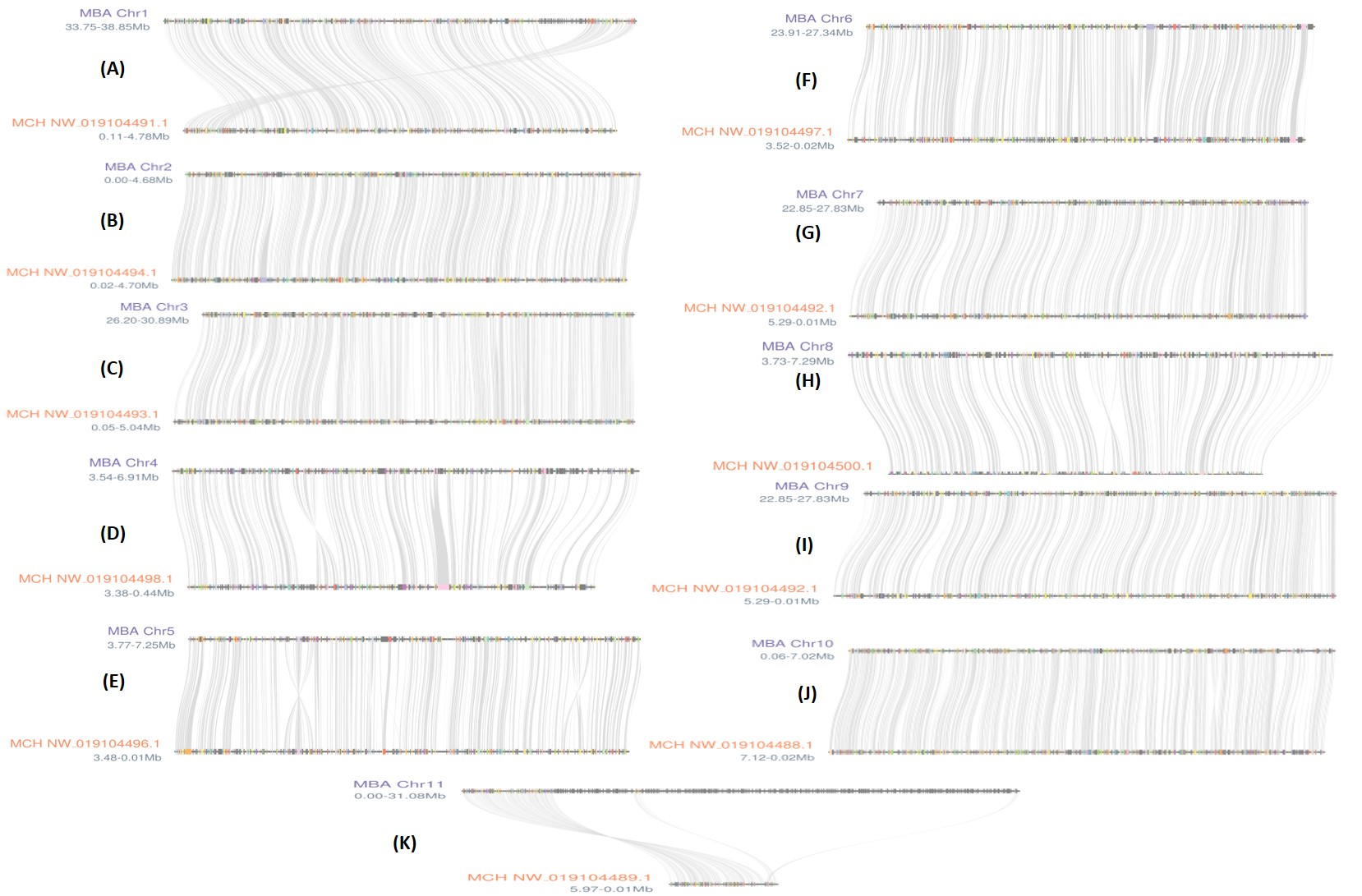


**Supplementary Figure 3**: Microsyntenic relationship depicting a collinear arrangement of homologous genes in genomic region of each of the 11 Chromosome (A-K) of *M. balsamina* (represented as MBA) with corresponding scaffolds *M. charantia* (represented as MCH)

**Supplementary Table 1:** Details of raw reads from different methods used in the study along with sequence length, GC% and SRA IDs from NCBI submission

| Sequencing method | Replicates | Reads | Sequence length | GC% |
| --- | --- | --- | --- | --- |
| 10x Genomics short reads (PE) | R1 | 23814179 | 150 | 39 |
|  | R2 | 37662141 | 150 | 39 |
|  | R3 | 34526672 | 150 | 39 |
|  | R4 | 15067110 | 150 | 39 |
| Oxford Nanopore reads | R1 | 2331456 | 37-222917 | 35 |
| HiC reads (PE) | R1 | 168098715 | 20-150 | 39 |

**Supplementary Table 2:** Maximum number of genes on each chromosome of *M. Balsamina* found homologous with genes on corresponding scaffolds of *M. charantia*

| *M. Balsamina* chromosomes | *M. charantia* scaffolds with maximum homologous genes | *M. Balsamina* genes |
| --- | --- | --- |
| Chr1 | NW_019104491.1 (scaffold 3) | 413 |
| Chr2 | NW_019104494.1 (scaffold 6) | 464 |
| Chr3 | NW_019104493.1 (scaffold 5) | 476 |
| Chr4 | NW_019104498.1 (scaffold 10) | 201 |
| Chr5 | NW_019104496.1 (scaffold 8) | 277 |
| Chr6 | NW_019104497.1 (scaffold 9) | 341 |
| Chr7 | NW_019104492.1 (scaffold 4) | 472 |
| Chr8 | NW_019104500.1 (scaffold 12) | 185 |
| Chr9 | NW_019104492.1 (scaffold 4) | 472 |
| Chr10 | NW_019104488.1 (scaffold 0) | 652 |
| Chr11 | NW_019104489.1 (scaffold 1) | 455 |
